# Network connectivity and structural correlates of survival in progressive supranuclear palsy and corticobasal syndrome

**DOI:** 10.1002/hbm.26342

**Published:** 2023-06-03

**Authors:** David J. Whiteside, Duncan Street, Alexander G. Murley, P. Simon Jones, Maura Malpetti, Boyd C. P. Ghosh, Ian Coyle‐Gilchrist, Alexander Gerhard, Michele T. Hu, Johannes C. Klein, P. Nigel Leigh, Alistair Church, David J. Burn, Huw R. Morris, James B. Rowe, Timothy Rittman

**Affiliations:** ^1^ Department of Clinical Neurosciences and Cambridge Centre for Parkinson‐plus University of Cambridge Cambridge UK; ^2^ Cambridge University Hospitals NHS Foundation Trust Cambridge UK; ^3^ Wessex Neurological Centre University Hospital Southampton Southampton UK; ^4^ Norfolk and Norwich University Hospital Norwich UK; ^5^ Division of Neuroscience and Experimental Psychology, Wolfson Molecular Imaging Centre University of Manchester Manchester UK; ^6^ Departments of Geriatric Medicine and Nuclear Medicine University of Duisburg‐Essen Duisburg Germany; ^7^ Oxford Parkinson's Disease Centre and Division of Neurology, Nuffield Department of Clinical Neurosciences University of Oxford Oxford UK; ^8^ Department of Neuroscience Brighton and Sussex Medical School Brighton UK; ^9^ Department of Neurology Royal Gwent Hospital Newport UK; ^10^ Faculty of Medical Sciences Newcastle University Newcastle UK; ^11^ Department of Clinical and Movement Neurosciences University College London, Queen Square Institute of Neurology London UK; ^12^ MRC Cognition and Brain Sciences Unit University of Cambridge Cambridge UK

**Keywords:** connectivity, corticobasal syndrome, fMRI, prediction, progressive supranuclear palsy, survival, tauopathies

## Abstract

There is a pressing need to understand the factors that predict prognosis in progressive supranuclear palsy (PSP) and corticobasal syndrome (CBS), with high heterogeneity over the poor average survival. We test the hypothesis that the magnitude and distribution of connectivity changes in PSP and CBS predict the rate of progression and survival time, using datasets from the Cambridge Centre for Parkinson‐plus and the UK National PSP Research Network (PROSPECT‐MR). Resting‐state functional MRI images were available from 146 participants with PSP, 82 participants with CBS, and 90 healthy controls. Large‐scale networks were identified through independent component analyses, with correlations taken between component time series. Independent component analysis was also used to select between‐network connectivity components to compare with baseline clinical severity, longitudinal rate of change in severity, and survival. Transdiagnostic survival predictors were identified using partial least squares regression for Cox models, with connectivity compared to patients' demographics, structural imaging, and clinical scores using five‐fold cross‐validation. In PSP and CBS, between‐network connectivity components were identified that differed from controls, were associated with disease severity, and were related to survival and rate of change in clinical severity. A transdiagnostic component predicted survival beyond demographic and motion metrics but with lower accuracy than an optimal model that included the clinical and structural imaging measures. Cortical atrophy enhanced the connectivity changes that were most predictive of survival. Between‐network connectivity is associated with variability in prognosis in PSP and CBS but does not improve predictive accuracy beyond clinical and structural imaging metrics.

## Graphical abstract

We tested whether MRI‐based biomarkers improve the prediction of survival time in progressive supranuclear palsy and corticobasal syndrome in two complementary datasets. We found that between‐network connectivity predicts variability in survival and progression in PSP and CBS but does not improve predictive accuracy beyond clinical and structural imaging metrics.

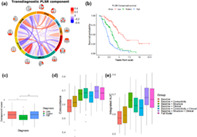

## 1 INTRODUCTION

Progressive supranuclear palsy (PSP) and corticobasal syndrome (CBS) are characterised by short average survival, but with significant variability in individual outcome (Chiu et al., 2010; Coyle‐Gilchrist et al., 2016). There is a pressing need to accurately predict rate of progression and survival time, to aid clinical management, assist stratification for clinical trials and to identify potential protective factors associated with better prognosis (Eimeren et al., 2019). Functional connectivity is a promising candidate to improve prognostication given the close association between functional organisation and changes in cognition with ageing and neurodegeneration (Chan et al., 2014; Rittman et al., 2019; Tsvetanov et al., 2021).

PSP and CBS are distinct clinical disorders in their classical forms but nonetheless overlap in their genetic risk factors, (Höglinger et al., 2011; Kouri et al., 2015) pathologies, (Kovacs, 2015) clinical features, (Armstrong et al., 2013; Höglinger, 2018; Höglinger et al., 2017; Murley et al., 2020) prognostic indicators, (Lansdall et al., 2019; Murley et al., 2021) and in current and potential therapeutic agents (Bluett et al., 2021; Boxer et al., 2020; VandeVrede et al., 2020). We make a distinction between the clinical syndromes of PSP and CBS, and the clinicopathological four‐repeat tauopathies of corticobasal degeneration (CBD) and PSP pathology. While the initial description of PSP, consisting of a supranuclear gaze palsy, axial predominant rigidity, and early falls, (Steele et al., 1964) is strongly predictive of PSP‐pathology, (Litvan, 1997; Osaki et al., 2004) highly varied clinical features are associated with the same proteinopathy (Dickson et al., 2011; Kovacs et al., 2020). The pathological aetiology of corticobasal syndrome is heterogeneous; PSP‐pathology is common as well as CBD (Koga et al., 2022). The presence of shared clinical features is recognised in the current diagnostic criteria for the clinical syndromes, (Armstrong et al., 2013; Höglinger et al., 2017) with operationalised definitions of PSP‐CBS, (Höglinger et al., 2017) CBD‐PSP, (Armstrong et al., 2013) and likely 4‐R tauopathy (Höglinger et al., 2017). Many patients with a clinical diagnosis of either PSP or CBS will have features of both diagnostic criteria (Murley et al., 2020). PSP and CBS have also been previously shown to share clinical determinants of survival, including apathy (Lansdall et al., 2019) and motor impairment (Murley et al., 2021). The degree of clinical convergence means that symptomatic therapeutic options are common between the two conditions (Bluett et al., 2021). Moreover, there is both growing interest in and completed examples of ‘basket’ designs for novel experimental agents, which recruit across the 4‐R tauopathies (Boxer et al., 2020; VandeVrede et al., 2020). Therefore, considering PSP and CBS together can aid prognostication, to gain understanding of how and when pathophysiological processes converge to determine outcome, and to generate mechanistic biomarkers relevant to both conditions.

Temporally correlated brain networks are consistently observed in healthy adults, across the lifespan, and can be identified by functional magnetic resonance imaging at rest (Beckmann et al., 2005; Biswal et al., 1995; Damoiseaux et al., 2006; Yeo et al., 2011). Altered functional organisation, representing dysfunctional neurons and networks, maybe a more sensitive measure of underlying disease state than regional atrophy or cross‐sectional performance on standardised clinical tasks. In neurodegenerative conditions network segregation is associated with maintained cognitive performance in the presence of pathology, (Ewers et al., 2021; Tsvetanov et al., 2021) with loss of network integrity and large‐scale network change occurring at the point of symptom onset (Rittman et al., 2019). It is therefore plausible that greater network disruption would imply poor longitudinal outcome. Resting state connectivity in neurodegeneration is influenced by inflammation, (Passamonti et al., 2019) synaptic loss, (Zhang et al., 2023) pathological proteins, (Cope et al., 2018; Franzmeier et al., 2022) white matter disease, (McColgan et al., 2017) neurotransmitter deficits, (Borchert et al., 2019; Klaassens et al., 2019) metabolism, (Sheline & Raichle, 2013) and cell death (Hampton et al., 2020). Identifying connectivity markers of survival would enable in vivo mechanistic testing of the importance of different components of the neurodegenerative cascade for outcome.

A challenge when assessing the impact of connectivity on survival is that even in healthy controls, individual connections show poor reproducibility and vary on repeat scanning (Lynch et al., 2020; Noble et al., 2019). However, multivariate data‐driven approaches to identify a small number of features, such as independent component analysis, significantly improve robustness of connectivity estimates (Elliott et al., 2018; Marek et al., 2022). This is important when considering the clinical syndromes of PSP and CBS where connectivity changes are diffuse, (Ballarini et al., 2020; Brown et al., 2017) in keeping with brain‐wide synaptic loss observed in vivo (Holland et al., 2020) and at post‐mortem (Bigio et al., 2001; Lipton et al., 2001). We, therefore, investigated the utility of functional connectivity to predict outcomes for individual diagnostic groups and transdiagnostically, adopting a whole‐brain approach rather than focusing on individual connections.

Data reduction techniques to identify common patterns of connectivity change may not give the most sensitive survival predictors. Machine learning approaches may be more successful in identifying predictors, but standard machine learning tools need to be modified when estimating time to death given the presence of censored data resulting from including individuals alive at the end of a follow‐up period (Spooner et al., 2020). The simpler partial least squares (PLS) regression for Cox models (Bastien, 2008; Bastien et al., 2005, 2015; Bertrand & Maumy‐Bertrand, 2021) provides a promising approach that is adapted to explain maximal variance in survival, identifies patterns using all features, and is suitable for high‐dimensional data.

We, therefore, used these methods to test whether connectivity changes are associated with poorer prognosis in PSP and CBS. We quantify connectivity through resting‐state functional MRI and compare the predictive value of connectivity with clinical metrics and structural imaging. To assess generalisation, we used *k*‐fold cross‐validation for data from two cohorts of PSP, CBS, and controls: from the Cambridge Centre for Parkinson‐plus (CCPP) and the UK National PSP Research Network (PROSPECT‐MR). We tested the following hypotheses: (i) between‐network connectivity differs between participants with neurodegeneration and controls; (ii) more extensive changes in connectivity predict faster clinical deterioration and shorter survival; and (iii) changes in connectivity provide additive information to predict prognosis beyond clinical and structural imaging measures.

## 2 METHODS

### 2.1 Participants

We recruited 146 participants with MDS‐PSP criteria probable or possible PSP, (Höglinger et al., 2017) 82 participants with the clinical phenotype of corticobasal syndrome, (Armstrong et al., 2013) and 90 age‐matched healthy controls from the CCPP and the PSP‐Corticobasal Syndrome‐Multiple System Atrophy‐UK (PROSPECT‐MR) study (Jabbari et al., 2020). Clinical assessments for the two cohorts included the PSP rating scale (PSPRS), (Golbe & Ohman‐Strickland, 2007) the Cambridge Behavioural Inventory‐Revised (CBIR) (Wear et al., 2008) and the Addenbrooke's Cognitive Examination‐Revised (ACER) (Mioshi et al., 2006). A total of 49 participants with PSP, 11 participants with CBS, and 9 healthy controls were excluded following assessment for motion (see below). We recorded survival and longitudinal neurocognitive assessments for participants up to 12 years from baseline imaging. We recorded date of death from participants’ NHS Summary Care Record. Demographic details and summary scores for included participants are described in Table 1.

**TABLE 1:** Demographic details for participants at baseline scan.

	Control (*n* = 81)	PSP (*n* = 97)	CBS (*n* = 71)	*F*/*t*/χ^2^	*p*
Scans (*n*)	94	118	88	‐	‐
Longitudinal imaging (*n*)	11	20	17		
Age (years)	68.5 (6.4)	70.1 (7.2)	67.9 (6.4)	2.1	.12
Sex (F/M)	46/35	43/54	42/29	4.5	.11
Number deceased	‐	70	40	‐	‐
Time to death (years)	‐	2.8 (1.8)	2.8 (2.0)	0.07	.95
3‐year survival (from the scan)	‐	42/87 (48%)	28/53 (53%)	0.12	.73
PSPRS *n* (%)	‐	35.3 (14.9) *85* (*88%*)	33.2 (15.8) *50* (*70%*)	−0.74	.46
CBIR *n* (%)	‐	44.4 (33.2) *67* (*69%*)	42.9 (25.8) *62* (*87%*)	−0.30	.76
ACER *n* (%)	‐	80.5 (14.3) *84* (*87%*)	75.2 (17.2) *66* (*93%*)	−2.0	0.048

*Note*: Continuous values are mean (SD). Group comparison used F or t‐test for groups with continuous data and chi‐squared for binary variables.

Abbreviations: ACER, Addenbrooke's cognitive examination‐revised, CBIR, Cambridge behavioural inventory revised; PSPRS, Progressive supranuclear palsy rating scale.

Twenty‐seven participants included with a clinical diagnosis of PSP proceeded to autopsy, with a predominant neuropathological diagnosis of PSP in 26, and 1 predominant argyrophilic grain disease. Sixteen of the participants included with corticobasal syndrome donated their brains. As expected in CBS, the underlying neuropathology was heterogenous with a final pathological diagnosis of CBD (*n* = 6), Alzheimer's disease (*n* = 5), mixed CBD/PSP (*n* = 1), PSP (*n* = 2), Pick's disease (*n* = 1) and multiple system atrophy (*n* = 1).

### 2.2 MRI acquisition and preprocessing

Participants at CCPP underwent fMRI imaging at 3T (TR 2–2.5 s, TE 30 ms, 3 × 3 × 3.5 mm/3 × 3 × 3.75 mm voxels, 140–305 volumes). High‐resolution T1‐weighted Magnetization Prepared Rapid Gradient Echo (MPRAGE) structural images (TR 2 s, TE 2.93 ms, flip angle 8°, voxel size 1.1 mm isotropic) were acquired during the same session for use in normalization. Participants from PROSPECT‐MR underwent a comparable fMRI imaging protocol at 3 T (TR 2.5 s, TE 30 ms, whole brain acquisition, 3 × 3 × 3.5 mm voxels, 200 volumes) and matched MPRAGE. A subset of 48 participants (20 PSP, 17 CBS, and 11 Controls) also had repeat imaging during the disease course (Table 1), with primary analysis from the baseline visit.

We adapted the FSL preprocessing pipeline (Smith et al., 2013) with the addition of wavelet despiking (Patel et al., 2014) given higher in‐scanner movement in participants with neurodegenerative diseases. For initial fMRI preprocessing the T1 structural images were cropped to remove non‐brain tissue followed by brain extraction using FSL's Brain Extraction Tool. We then used FSL's FEAT with the following steps: motion correction using MCFLIRT; spatial smoothing using a Gaussian kernel of 5 mm FWHW; grand‐mean intensity normalisation of the 4D dataset by a single multiplicative factor; and 100 Hz high‐pass temporal filtering. Structured artefacts were removed using independent component analysis denoising using FSL's MELODIC together with FIX, following hand‐training. Registration to high‐resolution structural and standard space images was carried out using FLIRT. Registration from high‐resolution structural to MNI space was then further refined using FNIRT nonlinear registration. Normalized images were inspected to ensure adequate registration. We did not use global signal regression, given the potential to remove neural signals and introduce anti‐correlations. (Murphy & Fox, 2017) Wavelet despiking was used for further removal of motion artefact.

Since in‐scanner participant motion in resting state fMRI has the potential to bias connectivity estimates, (Power et al., 2012) we excluded individuals above thresholds for metrics of in‐scanner motion (maximum spike percentage (Patel et al., 2014) of 40.8%, maximum framewise displacement (Power et al., 2012) of 5.7 mm, and maximum spatial standard deviation of successive volume differences (Smyser et al., 2010) of 10.3). Thresholds were derived from previously defined mean and standard deviation in a dataset of 408 fMRIs from controls and participants with neurodegenerative diseases, (Whiteside et al., 2021) taken as 1.2 standard deviations above the whole sample mean. Given that motion has relevant neural correlates (Geerligs et al., 2017) and likely relates to severity and survival in PSP and CBS, we did not include it as a covariate of no interest in our primary analysis. However, we additionally report the effect of adding mean framewise displacement, included it in our baseline model when comparing predictors of survival in disease, and report the effect of in‐scanner motion on survival. Summary motion indices by group for included participants are in Table S1.

### 2.3 Structural parcellation

We derived subcortical volumes and cortical thickness for parcels of the Brainnetome Atlas (Fan et al., 2016) using Freesurfer 7.1.0. (Dale et al., 1999). Subcortical volumes were adjusted for total intracranial volume by deriving residuals from linear regression between parcel volume and total intracranial volume (Voevodskaya et al., 2014). Volumes and thicknesses were averaged over the 48 larger regions and gyri to reduce number of features for model fitting. We additionally calculated volumes for four brainstem structures (medulla, pons, midbrain, and superior cerebellar peduncle) (Iglesias et al., 2015).

### 2.4 Between‐network connectivity

**FIGURE 1:**
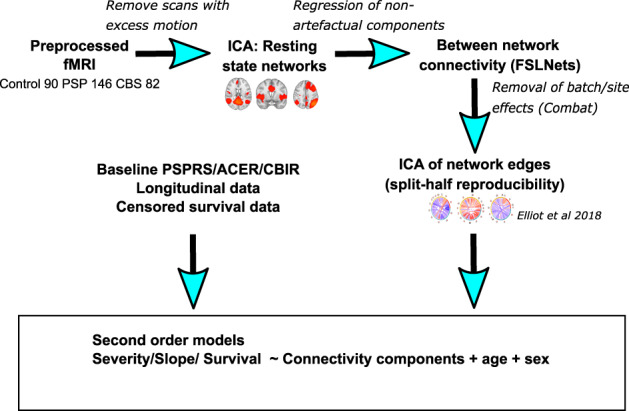
Pipeline for assessment of relationship between large‐scale network connectivity and severity, progression and survival Schematic representation of pipeline to derive independent components of between‐network connectivity to compare with outcome measures in PSP and CBS (ACER: Addenbrooke's cognitive examination‐revised, CBIR, Cambridge behavioural inventory revised; CBS, corticobasal syndrome; fMRI, functional magnetic resonance imaging; ICA, independent component analysis; PSP, progressive supranuclear palsy; PSPRS: progressive supranuclear palsy rating scale).

To identify between‐network connectivity patterns we employed the pipeline used by Elliot and colleagues (Elliott et al., 2018) (Figure 1). We adopted this approach as it captures multivariate large‐scale connectome patterns with improved test–retest reliability, important in these heterogenous conditions where widespread connectivity change and synaptic loss (Holland et al., 2020) suggest that isolated connections are unlikely to be reliably related to survival. Additionally, connections between large‐scale networks using group‐independent component analysis are more robust to spatial variability or differences between participants in alignment (Allen et al., 2012). We performed independent component analysis with a model order of 30 using FSL's MELODIC on preprocessed fMRI from patients and controls. These components were matched with their closest Yeo network (Yeo et al., 2011) using cross‐correlation against template maps and subsequent inspection. Components were selected if they were non‐artefactual and were a constituent of a Yeo network or overlapped with the thalamus. We did not include the Yeo limbic network given the influence of artefact and similarity to noise signal at 3‐Tesla fMRI, (Omidvarnia et al., 2021) and excluded inferior and ventral visual cortical regions due to the challenges in this region of differentiating BOLD signal from venous artefact (Boyd Taylor et al., 2019; Kay et al., 2019; Tsvetanov et al., 2015; Winawer et al., 2010). We then extracted component time series by regression of participant's preprocessed fMRI against the component maps, with time series for the chosen components taken forward for further analysis. Connectivity between components was calculated by full Pearson correlation between networks followed by Fisher r‐to‐Z normalization using FSLNets (Smith, Vidaurre, et al., 2013). We adjusted for scanner and site differences through an empirical Bayes framework using ComBat (Johnson et al., 2007; Yu et al., 2018). We compared the adjusted between‐component connectivity between patient groups and healthy controls in a linear model with age and sex as covariates of no interest, using the Benjamini‐Hochberg method (Benjamini & Hochberg, 1995) to control the false discovery rate.

We then performed a further independent component analysis (Hyvarinen, 1999) to identify a small number of components capturing between‐network connectivity patterns. We set a maximum model order of four since even in a large dataset only four components could be robustly inferred, (Elliott et al., 2018) using split‐half reproducibility of imaging component weights across subjects to determine the final number of components (Elliott et al., 2018).

### 2.5 Statistical approach—severity, progression, and survival

We took baseline imaging component scores for further analysis to compare between groups and correlate with severity, progression and survival. Age and sex were included as covariates of no interest in all models. Cross‐sectional analyses were performed using assessments at the earliest scanning date. *p* values were adjusted for multiple comparisons adjusted across components and neuropsychological tests (false discovery rate *p* < .05), with the corrected *p*‐value reported unless stated otherwise. All statistical analyses and visualization were performed in R (version 4.1.0) (Gu et al., 2014; Mowinckel, 2018; R Core Team, 2018).

To compare component scores between groups we performed a multivariate analysis of covariance. We compared clinical and neuropsychological markers of severity with scores for components of interest within a linear model, and test whether the disease groups differ in their component‐neuropsychological measure relationship through a refitted model including a group‐by‐component interaction.

A linear mixed‐effect model was used to calculate annual rates of changes in clinical and neuropsychological scores for participants with longitudinal data using the R package lme4 (Bates et al., 2015). Neuropsychological score was the dependent variable with years from baseline assessment as an independent variable. The model estimated a random intercept and slope to account for individual variability. The individual estimated slopes were included as a dependent variable in a second model with baseline connectivity component scores as predictors. Models were repeated with mean framewise displacement as a covariate of no interest. To assess whether connectivity components improve model fit for clinical progression (for PSPRS, CBIR, and ACER) beyond baseline severity, we performed stepwise regression using the Akaike information criteria. In the initial model, estimated slope was the dependent variable, with the two connectivity components, baseline clinical score, and total grey matter volume as independent variables. Age, sex, motion, and total intracranial volume were covariates of no interest, and not stepped out of the model.

We used a Cox proportional hazards regression analysis to assess the relationship between component score and time from scan until death with age and sex as covariates, an approach that enabled us to include participants alive at the end of the assessment period. Given the importance of in‐scanner motion as a potential confounder in quantifying connectivity, we additionally report the relationship between mean framewise displacement and time from scan until death.

### 2.6 Partial least squares for cox models

We proceeded to compare different potential predictors of survival in PSP and CBS. An independent component analysis finds statistically independent connectivity changes, but these may not be the best survival predictors. We, therefore, used PLS for Cox models (Bastien et al., 2005, 2015; Bertrand & Maumy‐Bertrand, 2021) to maximize covariance of the predictor to censored survival data. This finds broad connectomic patterns most predictive of survival and likely to improve reliability beyond focusing on individual connections.

We used a transdiagnostic approach with PLS regression for Cox models performed with all baseline patient scans as a single group. We derived models with different predictors to determine indicators of survival: connectivity patterns; structural imaging measures; and clinical scores. The PLS for Cox models approach also allow component scores to be calculated where there are missing data for clinical assessments, based on a modified non‐linear PLS algorithm where iterative regressions are performed with the available data (Bastien et al., 2005; Bertrand & Maumy‐Bertrand, 2021).

To determine the best survival predictors, we used 20 repeats of five‐fold cross‐validation comparing: a baseline model (age, sex, and mean framewise displacement); the baseline model combined with connectivity; the baseline model with structural measures of atrophy; the baseline model together with clinical scores (PSPRS, CBIR, and ACER); the baseline model with clinical scores and structural measures; the baseline model with clinical scores and connectivity; and a full model with all predictors. For each model, the number of components was chosen which maximised cross‐validation performance. We compared models using (i) concordance index, (Harrell, 1982) the proportion of pairs of participants where the hazards predicted by the model accord with observed survival, and (ii) area under the curve for survival data (Heagerty et al., 2000).

While partial least squares regression as a data reduction technique is designed to handle high‐dimensional data with multicollinearity, where collinearity is very high there is a risk of model misspecification and overfitting, (Bastien et al., 2015) particularly with a large number of predictors (Chun & Keleş, 2010). Since structural measures were found to have be strongly collinear, we compared the predictive accuracy and coefficients from the PLS regression with regularized regression for Cox models. We used an elastic net penalty with the *glmnet* function from the glmnet package (Friedman et al., 2010; Simon et al., 2011) in R. We performed 100 repeats of five‐fold cross‐validation, comparing mean concordance at the regularization term with highest cross‐validation performance with mean concordance from the PLS regression model.

On a *post‐hoc* basis, we repeated model comparison with PSP and CBS individually. We compared the same models as in our transdiagnostic assessment, with the addition of a combination of the baseline model with clinical scores and a composite of thalamic, pontine, and midbrain volume, given the risk of overfitting with higher feature number to participant ratio in these subgroups.

### 2.7 Baseline atrophy and longitudinal connectivity change

We tested whether baseline focal atrophy influenced the longitudinal change in connectomic predictors of survival for the subset of patients with repeat imaging. We first derived PLS connectivity component scores for scans after the baseline visit. We tested the relationship between connectivity and time from baseline imaging session in a linear mixed‐effect model with PLS connectivity component score as the dependent variable, time from baseline scan as a fixed effect, and a random intercept for each participant. We then refitted the model including an interaction term with time from baseline imaging and focal atrophy (mean cortical thickness or subcortical volume).

We proceeded to perform mediation analysis using the *mediation* (Tingley et al., 2014) package in R using bootstrapping with 100,000 draws, with the PLS connectivity component as a mediator, mean cortical thickness or subcortical volume as predictors and age, sex and the remaining atrophy marker as a covariate of no interest.

### 2.8 Data sharing

The PROSPECT‐MR dataset reported here may be available subject to a PROSPECT data‐sharing agreement, after review by the PROSPECT data access committee. For details on how to apply, please contact the senior authors. Data from the CCPP is available on reasonable request to the senior authors but may be subject to restrictions that protect confidentiality, and a data transfer agreement may be required according to the nature of the request.

## 3 RESULTS

### 3.1 Participants

We report results from the analysis from 97 participants with PSP, 71 participants with CBS, and 81 healthy controls, after data quality control. Demographic details at the baseline scan are in Table 1. There were no significant differences in age or sex, with a mean time to death under 3 years from baseline imaging in both diseases.

### 3.2 Between‐network connectivity

**FIGURE 2:**
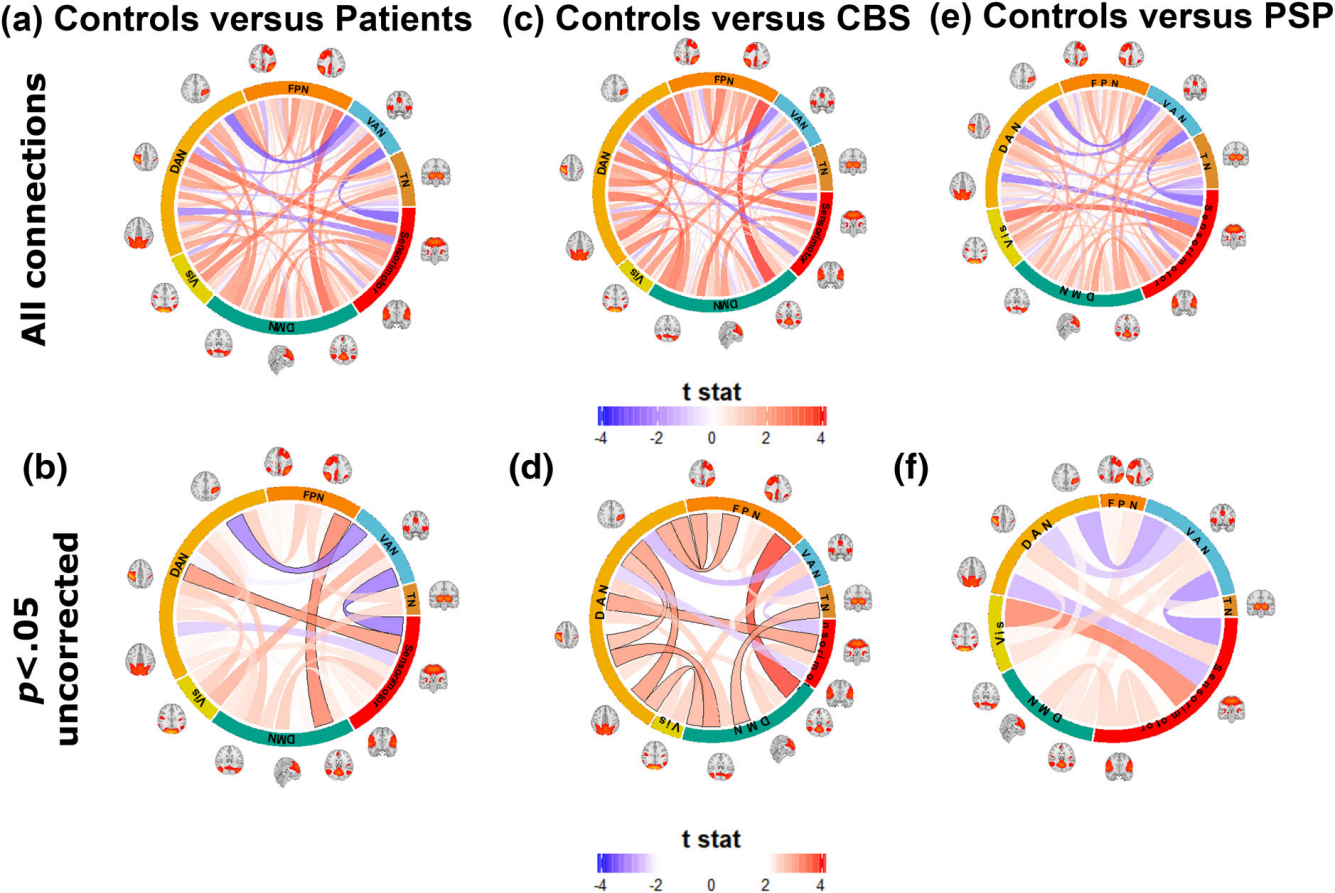
Between network connectivity in PSP and CBS. Differences in between‐network connectivity between all patients and controls (a) and (b), CBS and controls (c) and (d) and PSP and controls (e) and (f). Red links represent lower connectivity in patient groups, and blue links relatively increased connectivity versus controls. The bottom figures show only connections that show uncorrected significant differences (*p* < .05) between‐group differences beyond age and sex, with connections that remain significant after correction for multiple comparisons outlined in black.

Between‐network connectivity differences between patient groups and healthy controls are presented in Figure 2a, b. Comparing all patients to controls, connectivity was lower in patients for most between‐network connections, with significant reductions in connectivity in patients between sensorimotor and dorsal attention network regions and between default mode network and frontoparietal network components after correction for multiple comparisons. In the combined group with all patients, connectivity was significantly increased from the ventral attention network to dorsal attention and sensorimotor components.

Broadly similar connectivity differences from controls were observed in CBS (Figure 2c, d) and PSP (Figure 2e, f). In CBS we found reductions in connectivity between components of the dorsal attention network, the default mode network, and the frontoparietal network. Uncorrected increases in connectivity were found from the ventral attention network to dorsal attention and sensorimotor components, which were also observed when comparing PSP to controls. The largest reduction in connectivity in PSP was between the visual network and a component of the sensorimotor network. Comparing the disease groups, we found uncorrected greater reductions in connectivity in CBS predominantly in posterior components (including to regions of the dorsal attention network), with lower connectivity in PSP between the thalamus and a dorsal attention network component and between sensorimotor and visual regions (Figure S1). There were no significant differences between PSP and CBS after correction for multiple comparisons.

### 3.3 Structural metrics

Differences between groups in cortical thicknesses and subcortical volumes are presented in Figure S2. In PSP the greatest atrophy, when compared to controls, was found in subcortical regions and the frontal lobe. The largest effect sizes in CBS (vs. control participants) were in the frontal and parietal lobes, thalamus, and basal ganglia. Comparing PSP and CBS, atrophy was greater across cortical regions in CBS, with larger reductions in thalamic and brainstem volumes in PSP.

### 3.4 Connectivity relates to clinical severity

**FIGURE 3:**
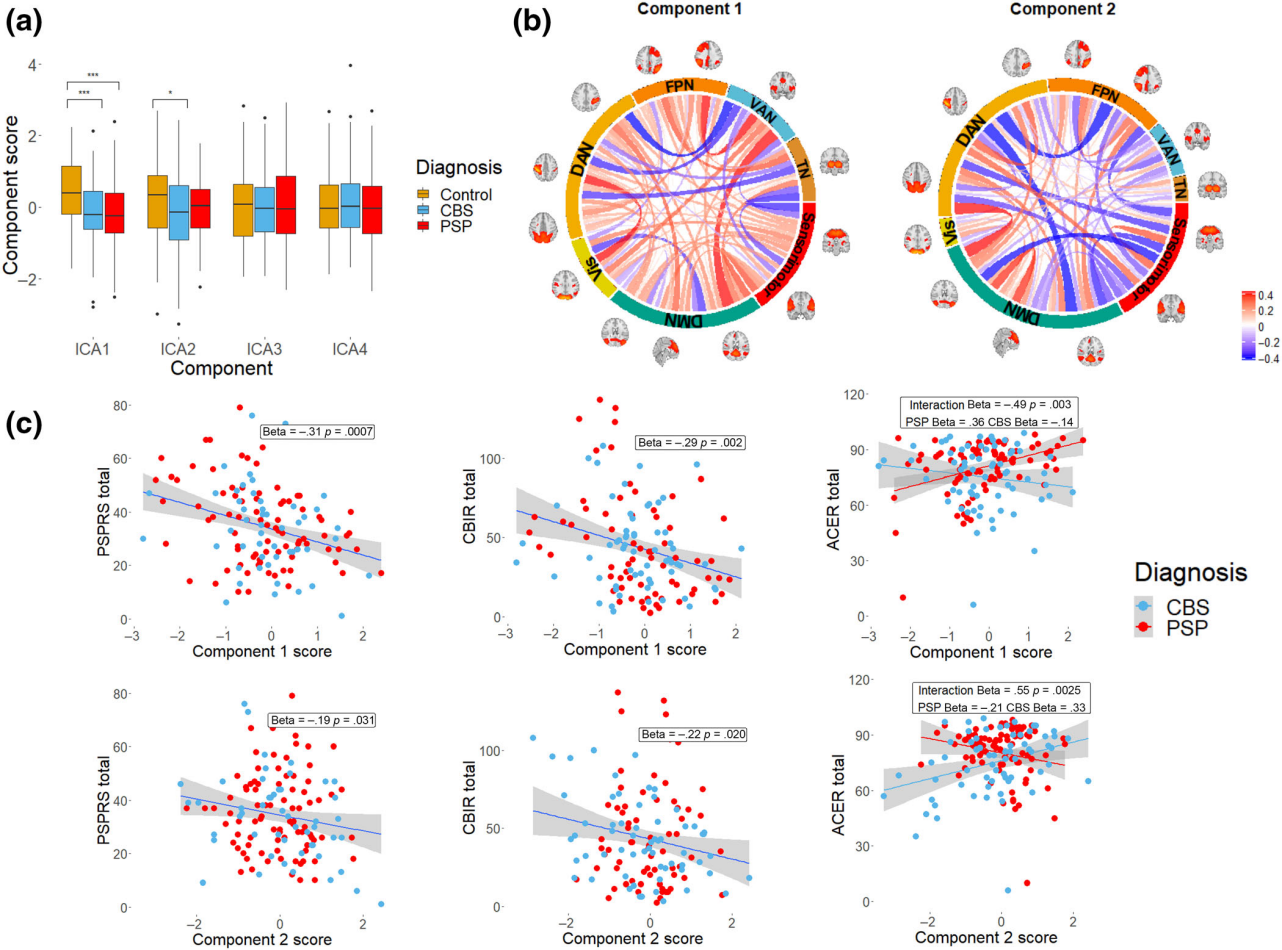
Between network connectivity and clinical severity in PSP and CBS. (a) Components were identified in PSP and CBS which differ between patients and controls, shown in (b). Connections represent the correlation between component score and edge so that for higher scoring subjects red indicates stronger connections and blue weaker. (c) Component scores correlate with clinical severity (ACER: Addenbrooke's cognitive examination‐revised; CBIR, Cambridge behavioural inventory revised; DAN, dorsal attention network; DMN, default mode network; FPN: frontoparietal network; PSPRS: progressive supranuclear palsy rating scale; TN: thalamic network; VAN, ventral attention network; Vis, visual).

We took the between‐network connections to an independent component analysis to capture broad patterns of connectivity to compare with clinical severity and progression. We found that using four components maximised split‐half reproducibility of component weights. Scores for the first component were decreased in both participants with PSP and CBS versus controls (Figure 3a *F* = 12.9, *p* = 2 × 10^−5^; PSP versus Control Tukey‐adjusted *p* = 2 × 10^−5^; CBS V Control Tukey‐adjusted *p* = .0002). Scores for the second component were decreased in CBS compared to controls, with no significant difference between either PSP and controls or between disease groups (*F* = 8.1, *p* = .01; PSP versus Control Tukey‐adjusted *p* = .2; CBS versus Control Tukey‐adjusted *p* = .014). The same components differed by group with mean framewise displacement included in the model (Component 1 *F* = 12.6, *p* = 3 × 10^−5^; Component 2 *F* = 4.9, *p* = .044). In Component 1 the disease state was associated with predominantly decreased connectivity but with increased connectivity between task‐positive, motor, and subcortical regions (Figure 3b). Lower scores in Component 2, as observed in CBS, were associated with relatively increased connectivity between the default mode, dorsal attention, and motor networks and decreased connectivity within these networks.

We considered the components that differed from controls in either disease group in subsequent analysis. Component 1 scores were associated with the PSPRS (Figure 3c Std Beta = −.31, *p* = .0007) and the CBIR (Std Beta = −.29, *p* = 0.002), with similar but weaker associations found with Component 2 (PSPRS Std Beta = −.19, *p* = .031; CBIR Std Beta = −.22 *p* = .020). The relationship between ACER and component scores differed between disease groups, with a significant interaction (Component 1 × diagnosis Interaction Std Beta = .49, *p* = .003, PSP Std Beta = .36, CBS Std Beta = −.14, Component 2 × diagnosis Interaction Std Beta = .55, *p* = .0025, PSP Std Beta = −.21 CBS Std Beta = 0.33), demonstrating the cognitive profile associated with greater posterior network involvement in CBS. With motion included in the model, these relationships remained significant, except for a marginal effect of the relationship between Component 2 and the PSPRS (Std Beta = −.17, *p* = .051) and the CBIR (Std Beta = −.19, *p* = .051).

### 3.5 Connectivity and disease progression

**FIGURE 4:**
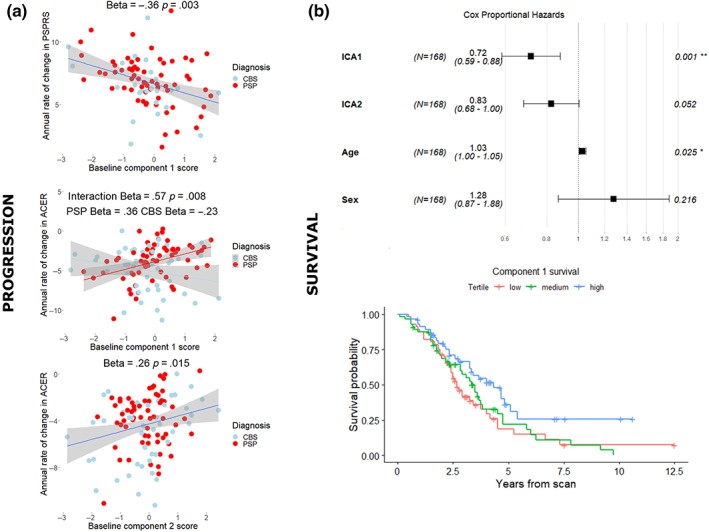
Connectivity predicts longitudinal survival in PSP and CBS. Component scores at baseline scan are associated with rate of change of severity (a) and are significantly associated with survival in a Cox proportional hazards model (b). For illustration, survival curves are shown by component scores divided into high, medium, and low‐scoring tertiles.

We tested whether baseline component scores were associated with a subsequent decline in neuropsychological assessments. Linear mixed‐effect models indicated an effect of time for all measures (Table S2). We found that baseline Component 1 score was associated with rate of progression in the PSPRS (Figure 4a Std Beta = −.36, *p* = 0.0006) and that baseline Component 2 score was associated with a greater rate of decline in the ACER (Std Beta = .26, *p* = .015). The implications of lower baseline Component 1 score on ACER varied by disease, with lower scores associated with a faster decline only in PSP (Component × diagnosis interaction Std Beta = .57, *p* = .008, PSP Std Beta = .36, CBS Std Beta = −.23). The relationships with Component 1 remained significant when mean framewise displacement was included in the model (PSPRS‐Component 1 Std Beta = −.36 *p* = .003; ACER‐Component 2 Std Beta = .22, FDR‐corrected *p* = .060, uncorrected *p* = .030; ACER‐Component 1 × diagnosis interaction *p* = .014). Lower Component 2 scores were also associated with an uncorrected increase in the rate of change of CBIR, including with adjustment for motion (Std Beta = −.21, uncorrected *p* = .044, FDR corrected *p* = .067).

We used stepwise regression to investigate if connectivity components were included in the best model of progression when incorporating baseline severity and total grey matter volume. For the PSPRS, Component 1 was included in the final model (Table S3), with Component 2 in the final model for ACER (Table S4), and both components for the CBIR (Table S5). Between‐network connectivity differences were associated with a more rapid decline in severity beyond baseline clinical scores and global atrophy.

### 3.6 Connectivity and survival

We found that a lower Component 1 score was a significant predictor of survival using Cox proportional hazards regression (Figure 4b Component 1 hazard ratio 0.72 CI 0.59–0.88 *p* = .001; Component 2 hazard ratio 0.83 CI 0.68–1.0 *p* = 0.052) in a model including age and sex as covariates. Component 1 remained a significant predictor with mean framewise displacement included in the model (Component 1 hazard ratio 0.73 CI 0.59–0.89 *p* = .002; Component 2 hazard ratio 0.87 CI 0.71–1.1 *p* = .19), an important consideration given that increased mean framewise displacement was associated with poorer survival in the whole cohort prior to exclusion for data quality (Figure S3). Significance remained with further addition of total grey matter volume and total intracranial volume to the model (Component 1 hazard ratio 0.74 CI 0.60–0.91 *p* = .005; Component 2 hazard ratio 0.89 CI 0.72–1.1 *p* = .26). The diagnosis by component interaction was not significant for either component.

### 3.7 Comparing transdiagnostic models to predict survival

We proceeded to investigate the optimal predictors of survival in patients with PSP and CBS. Since the most important connectivity changes for determining outcome may differ from the patterns of changes most common in disease, we used PLS for Cox models to maximise covariance between predictor and survival.

**FIGURE 5:**
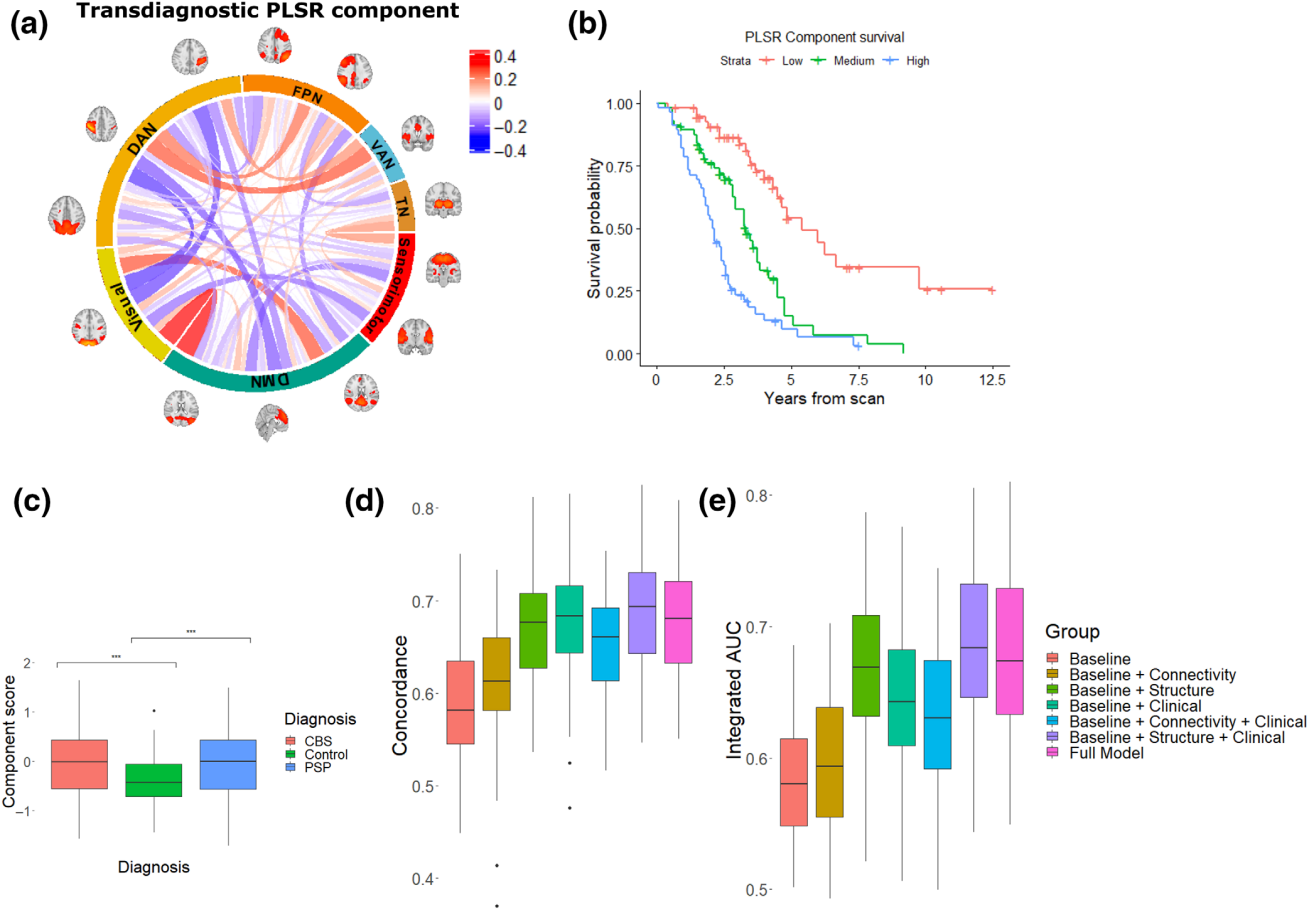
Identifying a transdiagnostic component predictive of outcome. We used partial least squares regression for Cox models to find a component (a) that maximised the covariance between connectivity and censored time to death. Connections represent PLSR weights, so that for higher scoring subjects red indicates stronger connections and blue weaker. This component did not differ between participants with PSP and those with CBS (c). Using five‐fold cross‐validation with outcome assessed using concordance analysis and integrated area under the curve, we found that connectivity provided additional information above patient's demographic information and inpatient motion, but with a combination of structural, clinical and baseline metrics providing the best predictive accuracy (d‐e). (DMN, default mode network; DAN, dorsal attention network; FPN, frontoparietal network; SM, sensorimotor; TN, thalamic network; VAN, ventral attention network).

We identified a connectivity component with covariance maximised to predict survival (Figure 5a), with worse survival related to relatively increased connectivity between task‐positive regions, from the thalamus to sensorimotor regions and from the default mode network to visual regions, representing a loss of segregation between these large‐scale networks, with decreased connectivity elsewhere. We found component scores differed between patient groups and controls (PSP versus Controls *t* = 3.8 Tukey‐adjusted *p* = .0005; CBS versus Control *t* = 3.6, *p* = .001), with no difference between PSP and CBS (Figure 5c *t* = 0.1, *p* = .99). We also identified two structural components predictive of survival (Figure S4). The highest absolute weights for the first component were for the thalamus, pons, and midbrain, with significant contributions from limbic and frontotemporal cortical regions. Lower scores in the second component, associated with worse survival, were for participants with thalamic and brainstem atrophy but relatively preserved cortical thickness. The highest component weight in a clinical component was for the PSPRS (Table S6).

We compared transdiagnostic predictive models of survival using repeat five‐fold cross‐validation to a baseline model consisting of age, sex, and mean framewise displacement from the fMRI scanning session since the latter is predictive of survival (Figure S2). We found that combining connectivity with the baseline model showed moderate improvement in predictive power, but that this was outperformed by both the combined baseline and structural model and the baseline and clinical models (Figures 5d, e Baseline: mean concordance 0.59, mean iAUC 0.58; Baseline + Connectivity: mean concordance 0.61, mean iAUC 0.59; Baseline + Structure mean concordance 0.67, mean iAUC 0.67; Baseline + Clinical mean concordance 0.68, mean iAUC 0.64). The best‐performing model combined baseline, structural and clinical metrics, while including all predictors in a single model worsened concordance (Baseline + Structural + Clinical mean concordance 0.68, mean iAUC 0.69; Baseline + Connectivity + Clinical mean concordance 0.65, mean iAUC 0.63; Full model mean concordance 0.68, mean iAUC 0.68). In all models including structural features best performance was with two PLS components, with one component for all other models.

We assessed the impact of collinearity on the PLS regression models, since high collinearity in regression may cause model misspecification or overfitting. Collinearity was low‐moderate between clinical, demographic, and connectivity measures (mean absolute Pearson's R 0.09, interquartile range 0.093, and maximum 0.52), and between structural and other measures (mean absolute Pearson's R 0.086, interquartile range 0.087, and maximum 0.48). Collinearity was greater between structural measures (mean absolute Pearson's R 0.40, interquartile range 0.32, and maximum 0.84). We, therefore, assessed whether regularised regression for Cox models for structural measures would improve predictive accuracy and specify different regional survival predictors. Predictive accuracy was unchanged between the PLS model and regularised regression with optimal regularisation term (PLSR mean concordance 0.67; regularised regression concordance 0.67). Regularised regression identified a highly similar anatomical distribution of structural survival predictors as in the PLS regression model, with largest coefficients in the pons and thalamus and non‐zero contributions from frontal and temporal regions.

To consider the potential impact of multiple collinear structural features we tested a further model with baseline and clinical measures, and the sum of volumes from the thalamus, pons, and midbrain. This *post‐hoc* model showed a modest improvement in performance over other models (mean concordance 0.7; mean iAUC 0.69).

We further tested survival predictors in each diagnostic group individually. In PSP, for a component derived using PLS regression with all predictors, highest weights were for the PSPRS, pons, midbrain, and thalamic volumes and bilateral superior temporal gyri thicknesses (Table S7). In CBS largest component weights were for the PSPRS, right thalamus, pons, and midbrain, with hippocampal atrophy also predictive of poor survival (Table S8). In addition, in CBS connectivity between posterior networks (posterior default mode network, dorsal attention, and visual) were also weighted highly. In both PSP and CBS best model performance was with baseline and clinical predictors, together with the composite thalamic, pons, and midbrain volume (PSP mean concordance 0.68, mean iAUC 0.68; CBS mean concordance 0.72 mean iAUC 0.69).

### 3.8 Focal atrophy and its relationship to connectivity

Since connectivity was only a moderate survival predictor, we investigated whether connectivity change may be driven by focal pathology. We considered the relationship between connectivity and cortical and subcortical atrophy, given that subcortical parcels had high loadings in the best survival model.

**FIGURE 6:**
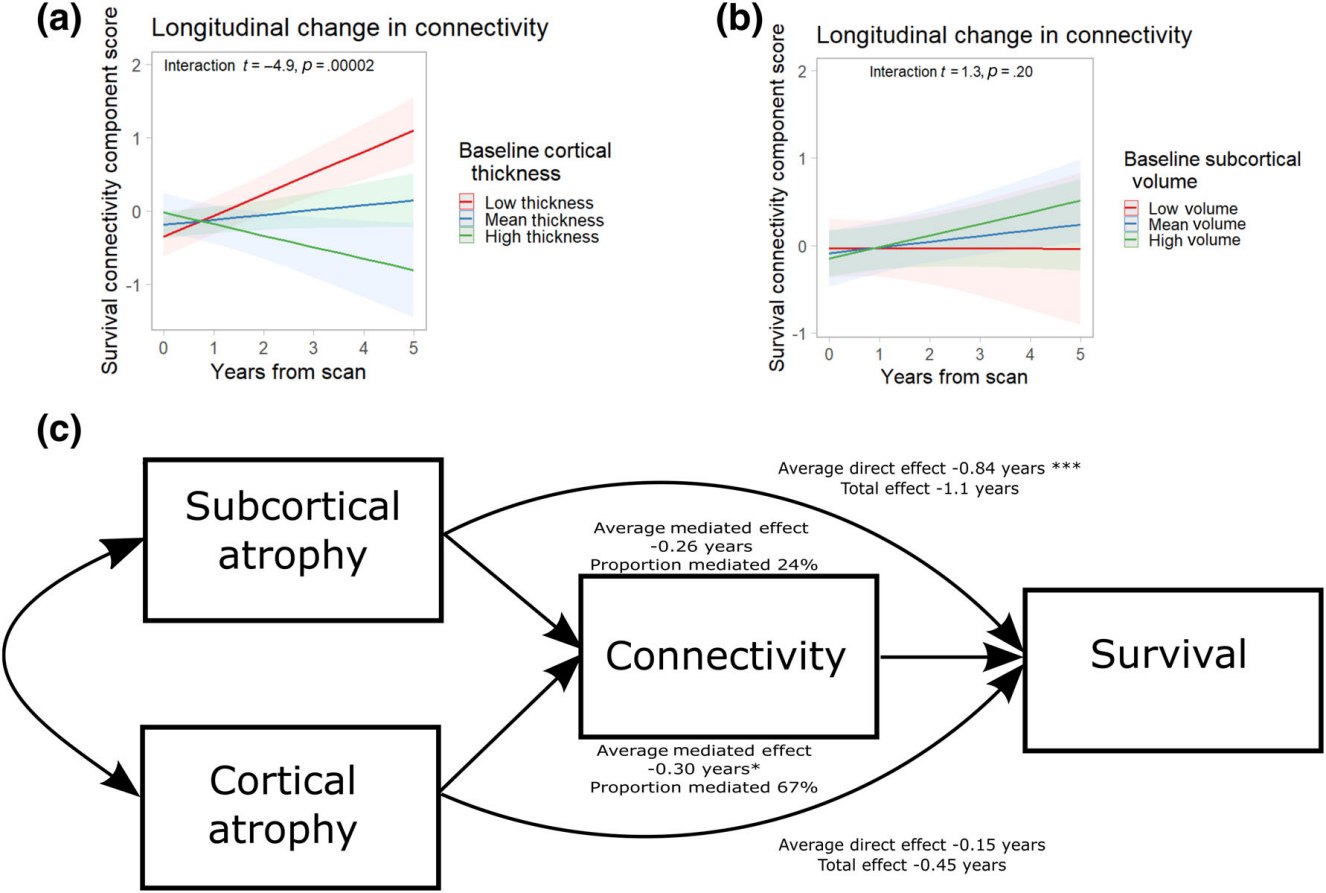
Connectome predictors of survival and regional atrophy. Baseline cortical atrophy (a) and not subcortical volume (b) is associated with longitudinal changes in connectivity predictive of survival. (c) Connectivity may mediate a significant proportion of the survival effect of cortical atrophy, while subcortical atrophy has a significant direct effect not mediated by connectivity. (*p < .05, **p < .01).

For individuals with longitudinal scanning, we found PLS component connectivity score increased over time (*t* = 2.7, *p* = .01), with higher component scores indicating worse survival. The rate of increase was greater in those with low cortical thickness (Figure 6a Cortical × years interaction *t* = −4.9, *p* = .0002), but not in those with reduced subcortical volume (Figure 6b interaction *t* = 1.3, *p* = .20). We then considered whether connectivity changes as identified in the PLSs regression may mediate the effect of atrophy on survival. We found that the connectivity component was a significant mediator of the effect of cortical atrophy on survival (average direct effect −0.15 years *p* = 0.51, average mediated effect −0.30 years *p* = 0.012, proportion mediated 67%), in contrast to the significant average direct effect of subcortical atrophy (average direct effect −0.84 years *p* = 0.0007, average mediated effect −0.26 years *p* = 0.057, proportion mediated 24%).

In summary, we have found that cortical rather than subcortical atrophy modulates the connectivity changes that are more strongly predictive of survival. However, the effects of subcortical atrophy on survival (primarily thalamic, pontine, and midbrain) are predominantly not mediated by changes in between‐network connectivity.

## 4 DISCUSSION

In this study of two independent cohorts, we have found that functional connectivity and focal atrophy predict disease trajectory for people with PSP and CBS, including their rate of progression and survival. There are connectivity changes associated with shorter time to death that are shared between the diseases, but these provide less robust predictions than simple clinical and structural imaging metrics. In the most accurate model for survival prediction, the greatest weights were for the PSPRS and thalamic, midbrain, and pontine volume. Cortical rather than subcortical volume at baseline was associated with subsequent progressive change in the functional connectivity that was predictive of survival. In contrast, the prognostic value of subcortical atrophy on survival is largely independent of the changes in network connectivity.

We found patterns of connectivity and structural change associated with poor survival that were shared between PSP and CBS. This is in keeping with the clinical, molecular, and pathological overlap between the diseases, (Höglinger, 2018; Murley et al., 2020) and implies the existence of common pathways important in determining survival. Commonality in survival predictors across diagnoses may arise through convergence in pathological involvement of structures important for survival. In our study, thalamic, pontine, and midbrain atrophy were key transdiagnostic survival predictors. Shared survival predictors may also occur at a network level, (Seeley, 2017) with similar patterns of network connectivity relevant to survival occurring in PSP and CBS despite differences in distribution of pathology. The accumulation of connectivity differences associated with poor survival over longitudinal imaging suggests active network change in the presence of pathology, rather than the identified patterns solely representing pre‐existing cognitive reserve (Stern et al., 2020).

The relationship between network connectivity and clinical severity is in keeping with findings that connectivity changes are closely associated with cognitive status in ageing (Chan et al., 2014) and in presymptomatic carriers of dementia‐causing mutations (Rittman et al., 2019; Tsvetanov et al., 2021). Our whole brain approach shows that connectivity changes that predict survival similarly represent a disruption to functional organisation rather than simply connectivity loss. Between‐network connectivity was predominantly decreased in participants with CBS and PSP, with increased connectivity also occurring across network hierarchies (Gotts et al., 2020; Margulies et al., 2016). Greater connectivity increased scores of a component with covariance maximised to predict survival, notably between task‐positive multimodal networks, from the thalamus to sensorimotor regions and from the default mode network to visual regions. The finding that relative regional increases in connectivity contribute to poor survival supports studies demonstrating an association between increased connectivity of higher cognitive networks in health and poor cognitive function, (Chan et al., 2014; Geerligs et al., 2017) and suggests that these connectivity differences indicate network inefficiency rather than compensatory changes. Cell death and the widespread cortical synaptic loss in PSP and CBS (Holland et al., 2020) may cause loss of segregation between distinct networks, such as the dorsal and ventral attention networks, with network segregation important in maintaining performance on cognitive tasks despite pathological change (Ewers et al., 2021; Tsvetanov et al., 2021). Functional brain organisation at rest relates to task‐based network changes (Cole et al., 2014, 2016). Altered connectivity between multimodal networks at rest in PSP and CBS may indicate task‐based network dysfunction, with behavioural and cognitive consequences relevant for disease progression (Lansdall et al., 2019; Murley et al., 2021).

Cortical atrophy and cortical network connectivity are interconnected, demonstrated by the finding that ‘epicenter’ regions of maximal atrophy can be used as seeds to select functional networks associated with neurodegenerative disease (Seeley et al., 2009; Zhou et al., 2012). Our findings support this observation, suggesting that connectivity change potentially mediates the survival effects of cortical atrophy. However, the largest effects on connectivity for structural measures were for the thalamus, pons, and midbrain. The importance of thalamic atrophy may be surprising given that in PSP cortical pathology defines the later stage of PSP tauopathy (Kovacs et al., 2020) while in CBS cortical rather than thalamic atrophy is a major imaging correlate (Boxer et al., 2006; Whitwell et al., 2010). The thalamus, pons, and midbrain contain fibres and nuclei important in diverse neuronal systems, (Roy et al., 2022) including in core motor functions that have been linked to survival in PSP and CBS (Glasmacher et al., 2017; Murley et al., 2021). While thalamocortical connections have been shown to be disrupted in primary tauopathies (Whitwell et al., 2011) our data suggest that the majority of the effect of subcortical atrophy on survival is not mediated by disruption to between‐network connectivity. Instead, the contribution of subcortical atrophy to survival is relatively independent of cortical atrophy or connectivity.

Our work highlights some of the barriers that limit between‐network connectivity from resting‐state functional MRI as a dementia biomarker. Network connectivity satisfies criteria for a biomarker of progression, anticipating clinical deterioration with a mechanistic rationale for a causal relationship (Eimeren et al., 2019). Yet even when adopting a methodology designed to increase reliability, the failure of connections to appear repeatedly in imaging means that results are insufficiently robust to provide accurate single‐subject survival predictions or to operate as an intermediate endpoint for clinical trials (D'Esposito, 2019). We selected a small number of independent components to assess between‐network connectivity, but this approach may fail to identify important functional connectivity or activation patterns relevant for survival. There is a range of alternative approaches to analysing functional data, including graph metric, dynamic connectivity, voxel‐wise, and gradient‐based analyses which may also capture characteristic differences predictive of survival. Further work is needed to determine whether these methods are more robust and with better test–retest reliability in neurodegenerative conditions with diffuse connectivity change and synaptic loss. One important consideration is the relevance of brainstem and thalamic structures in survival in PSP and CBS. Estimates of functional connectivity in these regions are affected by high physiological noise and other analytic approaches may be considered (Beissner et al., 2014).

There are other limitations to our study. We found that in‐scanner motion itself predicts survival in PSP and CBS. Despite adopting a principled preprocessing pipeline and not including motion confounds as a regressor in higher‐order regressions, (Geerligs et al., 2017) there is an inevitable compromise between over‐zealous preprocessing removing connectivity indicative of poor survival, and the failure to remove spurious connectivity deriving from motion (Power et al., 2012). To reduce the risk of motion biasing our assessments of connectivity we excluded significant numbers of participants, so it is possible that our conclusions do not apply to the excluded members of the cohort. We have used cross‐validation to assess the accuracy of our survival predictions across sites but have not tested results in a third, out‐of‐sample, cohort that varies by scanner and protocol (Yu et al., 2018). Although we present data from a sizeable cohort of participants, increasing study power would allow for model fine‐tuning and to compare machine learning approaches. We found only uncorrected differences between PSP and CBS and differential effects of connectivity on cognitive performance. We adopted an approach to analysis designed to detect diffuse changes in connectivity that might be associated with poor survival. Alternative methodological choices, such as completing analysis only with patient groups, may better capture between‐group differences and be useful to test if these differences are important in predicting survival. Recent developments (Horie et al., 2022) in fluid biomarkers may help improve in vivo prediction of pathological aetiology in tauopathies, which has the potential to assist prognostication.

In conclusion, between‐network functional brain connectivity predicts clinical deterioration and survival in PSP and CBS, with prediction in terms of cross‐validation and in terms of future changes after baseline scanning. However, functional connectivity provides less accurate predictions of survival than simpler measures of focal subcortical atrophy and baseline clinical severity.

## FUNDING INFORMATION

The study was co‐funded by the National Institute for Health Research (NIHR) Biomedical Research Centre at Cambridge University Hospitals NHS Foundation Trust and the University of Cambridge (NIHR203312), including their support for the Cambridge Brain Bank; the Cambridge Centre for Parkinson‐plus (RG95450); the Wellcome Trust (220258); the Evelyn Trust (17/09); National Institute for Health Research (ACF‐2018‐14‐016); the PSP Association UK (RG78738); Parkinson's UK; NIHR Oxford Biomedical Research Centre; NIHR Oxford Health Clinical Research Facility; Michael J Fox Foundation; NIHR University College London Hospitals (UCLH) Biomedical Research Centre; Race Against Dementia Alzheimer's Research UK (ARUK‐RADF2021A‐010); the Edmond J. Safra Philanthropic Foundation; the NIHR UCLH Clinical Research Facility; and Medical Research Council (SUAG/092168768; MC_UU_00030/14; MR/T033371/1). The views expressed are those of the authors and not necessarily those of the NIHR or the Department of Health and Social Care. For the purpose of open access, the author has applied a CC BY public copyright licence to any Author Accepted Manuscript version arising from this submission.

## CONFLICT OF INTEREST STATEMENT

James B. Rowe is a non‐remunerated trustee of the Guarantors of Brain, Darwin College, and the PSP Association; he provides consultancy to Alzheimer Research UK, Asceneuron, Biogen, Curasen, CumulusNeuro, UCB, SVHealth, and Wave, and has research grants from AZ‐Medimmune, Janssen, Lilly as industry partners in the Dementias Platform UK. Michele T. Hu received payment for Advisory Board attendance/consultancy for Biogen, Roche, CuraSen Therapeutics, Evidera, Manus Neurodynamica, and the MJFF Digital Health Assessment Board. Huw R. Morrisis employed by UCL. In the last 12 months, he reports paid consultancy from Roche and Amylyx; lecture fees/honoraria—BMJ, Kyowa Kirin, Movement Disorders Society. Research Grants from Parkinson's UK, Cure Parkinson's Trust, PSP Association, CBD Solutions, Drake Foundation, Medical Research Council, and Michael J Fox Foundation. Dr Morris is a co‐applicant on a patent application related to C9ORF72—Method for diagnosing a neurodegenerative disease (PCT/GB2012/052140). All other authors did not declare any funding sources that directly contributed to this study.

## Supporting information


**Data S1:** Supporting Information

## Data Availability

The PROSPECT‐MR dataset reported here may be available subject to a PROSPECT data sharing agreement, after review by the PROSPECT data access committee. For details of how to apply, please contact the senior authors. Data from the CCPP is available on reasonable request to the senior authors, but may be subject to restrictions that protect confidentiality or a data transfer agreement according to the nature of the request.

## References

[hbm26342-bib-0001] Allen, E. A. , Erhardt, E. B. , Wei, Y. , Eichele, T. , & Calhoun, V. D. (2012). Capturing inter‐subject variability with group independent component analysis of fMRI data: A simulation study. NeuroImage, 59(4), 4141–4159. 22019879 10.1016/j.neuroimage.2011.10.010 PMC3690335

[hbm26342-bib-0002] Armstrong, M. J. , Litvan, I. , Lang, A. E. , Bak, T. H. , Bhatia, K. P. , Borroni, B. , Boxer, A. L. , Dickson, D. W. , Grossman, M. , Hallett, M. , Josephs, K. A. , Kertesz, A. , Lee, S. E. , Miller, B. L. , Reich, S. G. , Riley, D. E. , Tolosa, E. , Troster, A. I. , Vidailhet, M. , & Weiner, W. J. (2013). Criteria for the diagnosis of corticobasal degeneration. Neurology, 80(5), 496–503. 23359374 10.1212/WNL.0b013e31827f0fd1 PMC3590050

[hbm26342-bib-0003] Ballarini, T. , Albrecht, F. , Mueller, K. , Jech, R. , Diehl‐Schmid, J. , Fliessbach, K. , Kassubek, J. , Lauer, M. , Fassbender, K. , Schneider, A. , Synofzik, M. , Wiltfang, J. , FTLD Consortium Germany , 4RTNI , Otto, M. , & Schroeter, M. L. (2020). Disentangling brain functional network remodeling in corticobasal syndrome—A multimodal MRI study. NeuroImage: Clinical, 25, 102112. 31821953 10.1016/j.nicl.2019.102112 PMC6906725

[hbm26342-bib-0004] Bastien, P. (2008). Deviance residuals based PLS regression for censored data in high dimensional setting. Chemometrics and Intelligent Laboratory Systems, 91(1), 78–86.

[hbm26342-bib-0005] Bastien, P. , Bertrand, F. , Meyer, N. , & Maumy‐Bertrand, M. (2015). Deviance residuals‐based sparse PLS and sparse kernel PLS regression for censored data. Bioinformatics, 31(3), 397–404. 25286920 10.1093/bioinformatics/btu660

[hbm26342-bib-0006] Bastien, P. , Vinzi, V. E. , & Tenenhaus, M. (2005). PLS generalised linear regression. Computational Statistics & Data Analysis, 48(1), 17–46.

[hbm26342-bib-0007] Bates, D. , Mächler, M. , Bolker, B. , & Walker, S. (2015). Fitting linear mixed‐effects models using lme4. Journal of Statistical Software, 67(1), 1–48. http://www.jstatsoft.org/v67/i01/

[hbm26342-bib-0008] Beckmann, C. F. , DeLuca, M. , Devlin, J. T. , & Smith, S. M. (2005). Investigations into resting‐state connectivity using independent component analysis. Philosophical Transactions of the Royal Society of London. Series B, Biological Sciences, 360(1457), 1001–1013. 16087444 10.1098/rstb.2005.1634 PMC1854918

[hbm26342-bib-0009] Beissner, F. , Schumann, A. , Brunn, F. , Eisenträger, D. , & Bär, K. J. (2014). Advances in functional magnetic resonance imaging of the human brainstem. NeuroImage, 86, 91–98. 23933038 10.1016/j.neuroimage.2013.07.081

[hbm26342-bib-0010] Benjamini, Y. , & Hochberg, Y. (1995). Controlling the false discovery rate: A practical and powerful approach to multiple testing. Journal of the Royal Statistical Society: Series B (Methodological)., 57(1), 289–300.

[hbm26342-bib-0011] Bertrand, F. , & Maumy‐Bertrand, M. (2021). Fitting and cross‐validating cox models to censored big data with missing values using extensions of partial least squares regression models. Frontiers in Big Data, 4, 91. 10.3389/fdata.2021.684794 PMC8591675 34790895

[hbm26342-bib-0012] Bigio, E. H. , Vono, M. B. , Satumtira, S. , Adamson, J. , Sontag, E. , Hynan, L. S. , White, C. L., III , Baker, M. , & Hutton, M. (2001). Cortical synapse loss in progressive Supranuclear palsy. Journal of Neuropathology and Experimental Neurology, 60(5), 403–410. 11379815 10.1093/jnen/60.5.403

[hbm26342-bib-0013] Biswal, B. , Zerrin Yetkin, F. , Haughton, V. M. , & Hyde, J. S. (1995). Functional connectivity in the motor cortex of resting human brain using echo‐planar MRI. Magnetic Resonance in Medicine, 34(4), 537–541. 8524021 10.1002/mrm.1910340409

[hbm26342-bib-0014] Bluett, B. , Pantelyat, A. Y. , Litvan, I. , Ali, F. , Apetauerova, D. , Bega, D. , Bloom, L. , Bower, J. , Boxer, A. L. , Dale, M. L. , Dhall, R. , Duquette, A. , Fernandez, H. H. , Fleisher, J. E. , Grossman, M. , Howell, M. , Kerwin, D. R. , Leegwater‐Kim, J. , Lepage, C. , … Golbe, L. I. (2021). Best practices in the clinical Management of progressive supranuclear palsy and corticobasal syndrome: A consensus statement of the CurePSP centers of care. Frontiers in Neurology, 1(12), 694872. 10.3389/fneur.2021.694872 PMC8284317 34276544

[hbm26342-bib-0015] Borchert, R. J. , Rittman, T. , Rae, C. L. , Passamonti, L. , Jones, S. P. , Vatansever, D. , Vázquez Rodríguez, P. , Ye, Z. , Nombela, C. , Hughes, L. E. , Robbins, T. W. , & Rowe, J. B. (2019). Atomoxetine and citalopram alter brain network organization in Parkinson's disease [Internet]. Brain Communications, 1(1), fcz013. 10.1093/braincomms/fcz013 31886460 PMC6924537

[hbm26342-bib-0016] Boxer, A. L. , Geschwind, M. D. , Belfor, N. , Gorno‐Tempini, M. L. , Schauer, G. F. , Miller, B. L. , Weiner, M. W. , & Rosen, H. J. (2006). Patterns of brain atrophy that differentiate Corticobasal degeneration syndrome from progressive Supranuclear palsy. Archives of Neurology, 63(1), 81–86. 16401739 10.1001/archneur.63.1.81

[hbm26342-bib-0017] Boxer, A. L. , Gold, M. , Feldman, H. , Boeve, B. F. , Dickinson, S. L. J. , Fillit, H. , Ho, C. , Paul, R. , Pearlman, R. , Sutherland, M. , Verma, A. , Arneric, S. P. , Alexander, B. M. , Dickerson, B. C. , Dorsey, E. R. , Grossman, M. , Huey, E. D. , Irizarry, M. C. , Marks, W. J. , … Tatton, N. (2020). New directions in clinical trials for frontotemporal lobar degeneration: Methods and outcome measures. Alzheimer's Dementia, 16(1), 131–143. 10.1016/j.jalz.2019.06.4956 PMC6949386 31668596

[hbm26342-bib-0018] Boyd Taylor, H. G. , Puckett, A. M. , Isherwood, Z. J. , & Schira, M. M. (2019). Vascular effects on the BOLD response and the retinotopic mapping of hV4. PLoS One, 14(6), e0204388. 31194745 10.1371/journal.pone.0204388 PMC6563965

[hbm26342-bib-0019] Brown, J. A. , Hua, A. Y. , Trujllo, A. , Attygalle, S. , Binney, R. J. , Spina, S. , Lee, S. E. , Kramer, J. H. , Miller, B. L. , Rosen, H. J. , Boxer, A. L. , & Seeley, W. W. (2017). Advancing functional dysconnectivity and atrophy in progressive supranuclear palsy. NeuroImage. Clinical, 16, 564–574. 28951832 10.1016/j.nicl.2017.09.008 PMC5605489

[hbm26342-bib-0020] Chan, M. Y. , Park, D. C. , Savalia, N. K. , Petersen, S. E. , & Wig, G. S. (2014). Decreased segregation of brain systems across the healthy adult lifespan. Proceedings of the National Academy of Sciences of the United States of America, 111(46), E4997–E5006. 25368199 10.1073/pnas.1415122111 PMC4246293

[hbm26342-bib-0021] Chiu, W. Z. , Kaat, L. D. , Seelaar, H. , Rosso, S. M. , Boon, A. J. , Kamphorst, W. , & van Swieten, J. C. (2010). Survival in progressive supranuclear palsy and frontotemporal dementia. Journal of Neurology, Neurosurgery & Psychiatry, 81(4), 441–445. 20360166 10.1136/jnnp.2009.195719

[hbm26342-bib-0022] Chun, H. , & Keleş, S. (2010). Sparse partial least squares regression for simultaneous dimension reduction and variable selection. Journal of the Royal Statistical Society Series B: Statistical Methodology., 72(1), 3–25. 20107611 10.1111/j.1467-9868.2009.00723.x PMC2810828

[hbm26342-bib-0023] Cole, M. W. , Bassett, D. S. , Power, J. D. , Braver, T. S. , & Petersen, S. E. (2014). Intrinsic and task‐evoked network architectures of the human brain. Neuron, 83, 238–251. 24991964 10.1016/j.neuron.2014.05.014 PMC4082806

[hbm26342-bib-0024] Cole, M. W. , Ito, T. , Bassett, D. S. , & Schultz, D. H. (2016). Activity flow over resting‐state networks shapes cognitive task activations. Nature Neuroscience, 19(12), 1718–1726. 27723746 10.1038/nn.4406 PMC5127712

[hbm26342-bib-0025] Cope, T. E. , Rittman, T. , Borchert, R. J. , Jones, P. S. , Vatansever, D. , Allinson, K. , Passamonti, L. , Vazquez Rodriguez, P. , Bevan‐Jones, W. R. , O'Brien, J. T. , & Rowe, J. B. (2018). Tau burden and the functional connectome in Alzheimer's disease and progressive supranuclear palsy. Brain, 141(2), 550–567. 29293892 10.1093/brain/awx347 PMC5837359

[hbm26342-bib-0026] Coyle‐Gilchrist, I. T. S. , Dick, K. M. , Patterson, K. , Vázquez Rodríquez, P. , Wehmann, E. , Wilcox, A. , Lansdall, C. J. , Dawson, K. E. , Wiggins, J. , Mead, S. , Brayne, C. , & Rowe, J. B. (2016). Prevalence, characteristics, and survival of frontotemporal lobar degeneration syndromes. Neurology, 86(18), 1736–1743. 27037234 10.1212/WNL.0000000000002638 PMC4854589

[hbm26342-bib-0027] Dale, A. M. , Fischl, B. , & Sereno, M. I. (1999). Cortical surface‐based analysis. I. Segmentation and surface reconstruction. Neuroimage, 9, 179–194. 9931268 10.1006/nimg.1998.0395

[hbm26342-bib-0028] Damoiseaux, J. S. , Rombouts, S. A. R. B. , Barkhof, F. , Scheltens, P. , Stam, C. J. , Smith, S. M. , & Beckmann, C. F. (2006). Consistent resting‐state networks across healthy subjects. Proceedings of the National Academy of Sciences of the United States of America, 103(37), 13848–13853. 16945915 10.1073/pnas.0601417103 PMC1564249

[hbm26342-bib-0029] D'Esposito, M. (2019). Are individual differences in human brain organization measured with functional MRI meaningful? Proceedings of the National Academy of Sciences of the United States of America, 116(45), 22432–22434. 31619565 10.1073/pnas.1915982116 PMC6842611

[hbm26342-bib-0030] Dickson, D. W. , Kouri, N. , Murray, M. E. , & Josephs, K. A. (2011). Neuropathology of frontotemporal lobar degeneration‐tau (FTLD‐tau). Journal of Molecular Neuroscience, 45(3), 384–389. 21720721 10.1007/s12031-011-9589-0 PMC3208128

[hbm26342-bib-0031] Eimeren, T. , Antonini, A. , Berg, D. , Bohnen, N. , Ceravolo, R. , Drzezga, A. , Höglinger, G. U. , Higuchi, M. , Lehericy, S. , Lewis, S. , Monchi, O. , Nestor, P. , Ondrus, M. , Pavese, N. , Peralta, M. C. , Piccini, P. , Pineda‐Pardo, J. Á. , Rektorová, I. , Rodríguez‐Oroz, M. , … MDS Neuroimaging Study Group and the JPND Working Group ASAP‐SynTau . (2019). Neuroimaging biomarkers for clinical trials in atypical parkinsonian disorders: Proposal for a neuroimaging biomarker utility system. Alzheimer's Dementia, 11(1), 301–309. 10.1016/j.dadm.2019.01.011 PMC6446052 30984816

[hbm26342-bib-0032] Elliott, L. T. , Sharp, K. , Alfaro‐Almagro, F. , Shi, S. , Miller, K. L. , Douaud, G. , Marchini, J. , & Smith, S. M. (2018). Genome‐wide association studies of brain imaging phenotypes in UK biobank. Nature, 562(7726), 210–216. 30305740 10.1038/s41586-018-0571-7 PMC6786974

[hbm26342-bib-0033] Ewers, M. , Luan, Y. , Frontzkowski, L. , Neitzel, J. , Rubinski, A. , Dichgans, M. , Hassenstab, J. , Gordon, B. A. , Chhatwal, J. P. , Levin, J. , Schofield, P. , Benzinger, T. L. S. , Morris, J. C. , Goate, A. , Karch, C. M. , Fagan, A. M. , McDade, E. , Allegri, R. , Berman, S. , … for the Alzheimer's Disease Neuroimaging Initiative and the Dominantly Inherited Alzheimer Network . (2021). Segregation of functional networks is associated with cognitive resilience in Alzheimer's disease. Brain, 144(7), 2176–2185. 33725114 10.1093/brain/awab112 PMC8370409

[hbm26342-bib-0034] Fan, L. , Li, H. , Zhuo, J. , Zhang, Y. , Wang, J. , Chen, L. , Yang, Z. , Chu, C. , Xie, S. , Laird, A. R. , Fox, P. T. , Eickhoff, S. B. , Yu, C. , & Jiang, T. (2016). The human brainnetome atlas: A new brain atlas based on connectional architecture. Cerebral Cortex, 26, 3508–3526. 27230218 10.1093/cercor/bhw157 PMC4961028

[hbm26342-bib-0035] Franzmeier, N. , Brendel, M. , Beyer, L. , Slemann, L. , Kovacs, G. G. , Arzberger, T. , Kurz, C. , Respondek, G. , Lukic, M. J. , Biel, D. , Rubinski, A. , Frontzkowski, L. , Hummel, S. , Müller, A. , Finze, A. , Palleis, C. , Joseph, E. , Weidinger, E. , Katzdobler, S. , … Ewers, M. (2022). Tau deposition patterns are associated with functional connectivity in primary tauopathies. Nature Communications, 13(1), 1362. 10.1038/s41467-022-28896-3 PMC8924216 35292638

[hbm26342-bib-0036] Friedman, J. , Hastie, T. , & Tibshirani, R. (2010). Regularization paths for generalized linear models via coordinate descent. Journal of Statistical Software, 33(1), 1–22. http://www.jstatsoft.org/v33/i01/ PMC2929880 20808728

[hbm26342-bib-0037] Geerligs, L. , Tsvetanov, K. A. , & Cam‐CAN, H. R. N. (2017). Challenges in measuring individual differences in functional connectivity using fMRI: The case of healthy aging: Measuring individual differences using fMRI. Human Brain Mapping, 38(8), 4125–4156. 28544076 10.1002/hbm.23653 PMC5518296

[hbm26342-bib-0038] Glasmacher, S. A. , Leigh, P. N. , & Saha, R. A. (2017). Predictors of survival in progressive supranuclear palsy and multiple system atrophy: A systematic review and meta‐analysis. Journal of Neurology, Neurosurgery, and Psychiatry, 88(5), 402–411. 28250027 10.1136/jnnp-2016-314956

[hbm26342-bib-0039] Golbe, L. I. , & Ohman‐Strickland, P. A. (2007). A clinical rating scale for progressive supranuclear palsy. Brain, 130(Pt 6), 1552–1565. 17405767 10.1093/brain/awm032

[hbm26342-bib-0040] Gotts, S. J. , Gilmore, A. W. , & Martin, A. (2020). Brain networks, dimensionality, and global signal averaging in resting‐state fMRI: Hierarchical network structure results in low‐dimensional spatiotemporal dynamics. NeuroImage, 205, 116289. 31629827 10.1016/j.neuroimage.2019.116289 PMC6919311

[hbm26342-bib-0041] Gu, Z. , Gu, L. , Eils, R. , Schlesner, M. , & Brors, B. (2014). Circlize implements and enhances circular visualization in R. Bioinformatics, 30(19), 2811–2812. 24930139 10.1093/bioinformatics/btu393

[hbm26342-bib-0042] Hampton, O. L. , Buckley, R. F. , Manning, L. K. , Scott, M. R. , Properzi, M. J. , Peña‐Gómez, C. , Jacobs, H. I. L. , Chhatwal, J. P. , Johnson, K. A. , Sperling, R. A. , & Schultz, A. P. (2020). Resting‐state functional connectivity and amyloid burden influence longitudinal cortical thinning in the default mode network in preclinical Alzheimer's disease. NeuroImage: Clinical, 28, 102407. 32942175 10.1016/j.nicl.2020.102407 PMC7498941

[hbm26342-bib-0043] Harrell, F. E. (1982). Evaluating the yield of medical tests. Journal of the American Medical Association, 247(18), 2543. 7069920

[hbm26342-bib-0044] Heagerty, P. J. , Lumley, T. , & Pepe, M. S. (2000). Time‐dependent ROC curves for censored survival data and a diagnostic marker. Biometrics, 56(2), 337–344. 10877287 10.1111/j.0006-341x.2000.00337.x

[hbm26342-bib-0046] Höglinger, G. U. (2018). Is it useful to classify progressive Supranuclear palsy and Corticobasal degeneration as different disorders? No. Movement Disorders Clinical Practice, 5(2), 141–144. 30363409 10.1002/mdc3.12582 PMC6174469

[hbm26342-bib-0047] Höglinger, G. U. , Melhem, N. M. , Dickson, D. W. , Sleiman, P. M. A. , Wang, L. S. , Klei, L. , Rademakers, R. , de Silva, R. , Litvan, I. , Riley, D. E. , van Swieten, J. C. , Heutink, P. , Wszolek, Z. K. , Uitti, R. J. , Vandrovcova, J. , Hurtig, H. I. , Gross, R. G. , Maetzler, W. , Goldwurm, S. , … Schellenberg, G. D. (2011). Identification of common variants influencing risk of the tauopathy progressive supranuclear palsy. Nature Genetics, 43(7), 699–705. 21685912 10.1038/ng.859 PMC3125476

[hbm26342-bib-0048] Höglinger, G. U. , Respondek, G. , Stamelou, M. , Kurz, C. , Josephs, K. A. , Lang, A. E. , Mollenhauer, B. , Müller, U. , Nilsson, C. , Whitwell, J. L. , Arzberger, T. , Englund, E. , Gelpi, E. , Giese, A. , Irwin, D. J. , Meissner, W. G. , Pantelyat, A. , Rajput, A. , van Swieten, J. C. , … for the Movement Disorder Society‐endorsed PSP Study Group . (2017). Clinical diagnosis of progressive supranuclear palsy: The movement disorder society criteria. Movement Disorders, 32(6), 853–864. 28467028 10.1002/mds.26987 PMC5516529

[hbm26342-bib-0049] Holland, N. , Jones, P. S. , Savulich, G. , Wiggins, J. K. , Hong, Y. T. , Fryer, T. D. , Manavaki, R. , Sephton, S. M. , Boros, I. , Malpetti, M. , Hezemans, F. H. , Aigbirhio, F. I. , Coles, J. P. , O'Brien, J. , & Rowe, J. B. (2020). Synaptic loss in primary tauopathies revealed by [ ^ 11 ^ c ] ucb‐j positron emission tomography. Movement Disorders, 35(10), 1834–1842. 32652635 10.1002/mds.28188 PMC7611123

[hbm26342-bib-0050] Horie, K. , Barthélemy, N. R. , Spina, S. , VandeVrede, L. , He, Y. , Paterson, R. W. , Wright, B. A. , Day, G. S. , Davis, A. A. , Karch, C. M. , Seeley, W. W. , Perrin, R. J. , Koppisetti, R. K. , Shaikh, F. , Lago, A. L. , Heuer, H. W. , Ghoshal, N. , Gabelle, A. , Miller, B. L. , … Sato, C. (2022). CSF tau microtubule‐binding region identifies pathological changes in primary tauopathies. Nature Medicine, 28, 2547–2554. https://www.nature.com/articles/s41591-022-02075-9 10.1038/s41591-022-02075-9 PMC9800273 36424467

[hbm26342-bib-0051] Hyvarinen, A. (1999). Fast and robust fixed‐point algorithms for independent component analysis. IEEE Transactions on Neural Networks, 10(3), 626–634. 18252563 10.1109/72.761722

[hbm26342-bib-0052] Iglesias, J. E. , van Leemput, K. , Bhatt, P. , Casillas, C. , Dutt, S. , Schuff, N. , Truran‐Sacrey, D. , Boxer, A. , Fischl, B. , & Alzheimer's Disease Neuroimaging Initiative . (2015). Bayesian segmentation of brainstem structures in MRI. NeuroImage, 113, 184–195. 25776214 10.1016/j.neuroimage.2015.02.065 PMC4434226

[hbm26342-bib-0053] Jabbari, E. , Holland, N. , Chelban, V. , Jones, P. S. , Lamb, R. , Rawlinson, C. , Guo, T. , Costantini, A. A. , Tan, M. M. X. , Heslegrave, A. J. , Roncaroli, F. , Klein, J. C. , Ansorge, O. , Allinson, K. S. J. , Jaunmuktane, Z. , Holton, J. L. , Revesz, T. , Warner, T. T. , Lees, A. J. , … Morris, H. R. (2020). Diagnosis across the Spectrum of progressive Supranuclear palsy and Corticobasal syndrome. JAMA Neurology, 77(3), 377–387. 31860007 10.1001/jamaneurol.2019.4347 PMC6990759

[hbm26342-bib-0054] Johnson, W. E. , Li, C. , & Rabinovic, A. (2007). Adjusting batch effects in microarray expression data using empirical Bayes methods. Biostatistics, 8(1), 118–127. 16632515 10.1093/biostatistics/kxj037

[hbm26342-bib-0055] Kay, K. , Jamison, K. W. , Vizioli, L. , Zhang, R. , Margalit, E. , & Ugurbil, K. (2019). A critical assessment of data quality and venous effects in sub‐millimeter fMRI. NeuroImage, 189, 847–869. 30731246 10.1016/j.neuroimage.2019.02.006 PMC7737092

[hbm26342-bib-0056] Klaassens, B. L. , van Gerven, J. M. A. , Klaassen, E. S. , van der Grond, J. , & Rombouts, S. A. R. B. (2019). Cholinergic and serotonergic modulation of resting state functional brain connectivity in Alzheimer's disease. NeuroImage, 199, 143–152. 31112788 10.1016/j.neuroimage.2019.05.044

[hbm26342-bib-0057] Koga, S. , Josephs, K. A. , Aiba, I. , Yoshida, M. , & Dickson, D. W. (2022). Neuropathology and emerging biomarkers in corticobasal syndrome. Journal of Neurology, Neurosurgery, and Psychiatry, 93(9), 919–929. 35697501 10.1136/jnnp-2021-328586 PMC9380481

[hbm26342-bib-0058] Kouri, N. , Ross, O. A. , Dombroski, B. , Younkin, C. S. , Serie, D. J. , Soto‐Ortolaza, A. , Baker, M. , Finch, N. C. A. , Yoon, H. , Kim, J. , Fujioka, S. , McLean, C. A. , Ghetti, B. , Spina, S. , Cantwell, L. B. , Farlow, M. R. , Grafman, J. , Huey, E. D. , Ryung Han, M. , … Dickson, D. W. (2015). Genome‐wide association study of corticobasal degeneration identifies risk variants shared with progressive supranuclear palsy. Nature Communications, 6(1), 7247. 10.1038/ncomms8247 PMC4469997 26077951

[hbm26342-bib-0059] Kovacs, G. G. (2015). Invited review: Neuropathology of tauopathies: Principles and practice: Neuropathology of tauopathies. Neuropathology and Applied Neurobiology, 41(1), 3–23. 25495175 10.1111/nan.12208

[hbm26342-bib-0060] Kovacs, G. G. , Lukic, M. J. , Irwin, D. J. , Arzberger, T. , Respondek, G. , Lee, E. B. , Coughlin, D. , Giese, A. , Grossman, M. , Kurz, C. , McMillan, C. T. , Gelpi, E. , Compta, Y. , van Swieten, J. C. , Laat, L. D. , Troakes, C. , al‐Sarraj, S. , Robinson, J. L. , Roeber, S. , … Höglinger, G. U. (2020). Distribution patterns of tau pathology in progressive supranuclear palsy. Acta Neuropathologica, 140(2), 99–119. 32383020 10.1007/s00401-020-02158-2 PMC7360645

[hbm26342-bib-0061] Lansdall, C. J. , Coyle‐Gilchrist, I. T. S. , Vázquez Rodríguez, P. , Wilcox, A. , Wehmann, E. , Robbins, T. W. , & Rowe, J. B. (2019). Prognostic importance of apathy in syndromes associated with frontotemporal lobar degeneration. Neurology, 92(14), e1547–e1557. 30842292 10.1212/WNL.0000000000007249 PMC6448451

[hbm26342-bib-0062] Lipton, A. M. , Cullum, C. M. , Satumtira, S. , Sontag, E. , Hynan, L. S. , White, C. L III. , & Bigio, E. H. (2001). Contribution of asymmetric synapse loss to lateralizing clinical deficits in frontotemporal dementias. Archives of Neurology, 58(8), 1233–1239. 11493163 10.1001/archneur.58.8.1233

[hbm26342-bib-0063] Litvan, I. (1997). Which clinical features differentiate progressive supranuclear palsy (Steele–Richardson–Olszewski syndrome) from related disorders? A clinicopathological study. Brain, 120(1), 65–74. 9055798 10.1093/brain/120.1.65

[hbm26342-bib-0064] Lynch, C. J. , Power, J. D. , Scult, M. A. , Dubin, M. , Gunning, F. M. , & Liston, C. (2020). Rapid precision functional mapping of individuals using multi‐Echo fMRI. Cell Reports, 33(12), 108540. 33357444 10.1016/j.celrep.2020.108540 PMC7792478

[hbm26342-bib-0065] Marek, S. , Tervo‐Clemmens, B. , Calabro, F. J. , Montez, D. F. , Kay, B. P. , Hatoum, A. S. , Donohue, M. R. , Foran, W. , Miller, R. L. , Hendrickson, T. J. , Malone, S. M. , Kandala, S. , Feczko, E. , Miranda‐Dominguez, O. , Graham, A. M. , Earl, E. A. , Perrone, A. J. , Cordova, M. , Doyle, O. , … Dosenbach, N. U. F. (2022). Reproducible brain‐wide association studies require thousands of individuals. Nature, 603(7902), 654–660. 35296861 10.1038/s41586-022-04492-9 PMC8991999

[hbm26342-bib-0066] Margulies, D. S. , Ghosh, S. S. , Goulas, A. , Falkiewicz, M. , Huntenburg, J. M. , Langs, G. , Bezgin, G. , Eickhoff, S. B. , Castellanos, F. X. , Petrides, M. , Jefferies, E. , & Smallwood, J. (2016). Situating the default‐mode network along a principal gradient of macroscale cortical organization. Proceedings of the National Academy of Sciences of the United States of America, 113(44), 12574–12579. 27791099 10.1073/pnas.1608282113 PMC5098630

[hbm26342-bib-0067] McColgan, P. , Gregory, S. , Razi, A. , Seunarine, K. K. , Gargouri, F. , Durr, A. , Roos, R. A. , Leavitt, B. R. , Scahill, R. I. , Clark, C. A. , Tabrizi, S. J. , Rees, G. , Track On‐HD Investigators , Coleman, A. , Decolongon, J. , Fan, M. , Petkau, T. , Jauffret, C. , Justo, D. , … Johnson, H. (2017). White matter predicts functional connectivity in premanifest Huntington's disease. Annals of Clinical Translational Neurology, 4(2), 106–118. 28168210 10.1002/acn3.384 PMC5288460

[hbm26342-bib-0068] Mioshi, E. , Dawson, K. , Mitchell, J. , Arnold, R. , & Hodges, J. R. (2006). The Addenbrooke's cognitive examination revised (ACE‐R): A brief cognitive test battery for dementia screening. International Journal of Geriatric Psychiatry, 21(11), 1078–1085. 16977673 10.1002/gps.1610

[hbm26342-bib-0069] Mowinckel, A. (2018). Circular plots in R and adding images [Internet]. https://drmowinckels.io/blog/2018‐05‐25‐circluar‐plots‐in‐r‐and‐adding‐images/

[hbm26342-bib-0070] Murley, A. G. , Coyle‐Gilchrist, I. , Rouse, M. A. , Jones, P. S. , Li, W. , Wiggins, J. , Lansdall, C. , Rodríguez, P. V. , Wilcox, A. , Tsvetanov, K. A. , Patterson, K. , Lambon Ralph, M. A. , & Rowe, J. B. (2020). Redefining the multidimensional clinical phenotypes of frontotemporal lobar degeneration syndromes. Brain, 143(5), 1555–1571. 32438414 10.1093/brain/awaa097 PMC7241953

[hbm26342-bib-0071] Murley, A. G. , Rouse, M. A. , Coyle‐Gilchrist, I. T. S. , Jones, P. S. , Li, W. , Wiggins, J. , Lansdall, C. , Vázquez Rodríguez, P. , Wilcox, A. , Patterson, K. , & Rowe, J. B. (2021). Predicting loss of independence and mortality in frontotemporal lobar degeneration syndromes. Journal of Neurology, Neurosurgery, and Psychiatry, 92(7), 737–744. 33563798 10.1136/jnnp-2020-324903 PMC8223632

[hbm26342-bib-0072] Murphy, K. , & Fox, M. D. (2017). Towards a consensus regarding global signal regression for resting state functional connectivity MRI. NeuroImage, 154, 169–173. 27888059 10.1016/j.neuroimage.2016.11.052 PMC5489207

[hbm26342-bib-0073] Noble, S. , Scheinost, D. , & Constable, R. T. (2019). A decade of test‐retest reliability of functional connectivity: A systematic review and meta‐analysis. NeuroImage, 203, 116157. 31494250 10.1016/j.neuroimage.2019.116157 PMC6907736

[hbm26342-bib-0074] Omidvarnia, A. , Zalesky, A. , Mansour, L. S. , van de Ville, D. , Jackson, G. D. , & Pedersen, M. (2021). Temporal complexity of fMRI is reproducible and correlates with higher order cognition. NeuroImage, 230, 117760. 33486124 10.1016/j.neuroimage.2021.117760

[hbm26342-bib-0075] Osaki, Y. , Ben‐Shlomo, Y. , Lees, A. J. , Daniel, S. E. , Colosimo, C. , Wenning, G. , & Quinn, N. (2004). Accuracy of clinical diagnosis of progressive supranuclear palsy. Movement Disorders, 19(2), 181–189. 14978673 10.1002/mds.10680

[hbm26342-bib-0076] Passamonti, L. , Tsvetanov, K. A. , Jones, P. S. , Bevan‐Jones, W. R. , Arnold, R. , Borchert, R. J. , Mak, E. , Su, L. , O'Brien, J. T. , & Rowe, J. B. (2019). Neuroinflammation and functional connectivity in Alzheimer's disease: Interactive influences on cognitive performance. The Journal of Neuroscience, 39(36), 7218–7226. 31320450 10.1523/JNEUROSCI.2574-18.2019 PMC6733539

[hbm26342-bib-0077] Patel, A. X. , Kundu, P. , Rubinov, M. , Jones, P. S. , Vértes, P. E. , Ersche, K. D. , Suckling, J. , & Bullmore, E. T. (2014). A wavelet method for modeling and despiking motion artifacts from resting‐state fMRI time series. NeuroImage, 95, 287–304. 24657353 10.1016/j.neuroimage.2014.03.012 PMC4068300

[hbm26342-bib-0078] Power, J. D. , Barnes, K. A. , Snyder, A. Z. , Schlaggar, B. L. , & Petersen, S. E. (2012). Spurious but systematic correlations in functional connectivity MRI networks arise from subject motion. NeuroImage, 59(3), 2142–2154. 22019881 10.1016/j.neuroimage.2011.10.018 PMC3254728

[hbm26342-bib-0079] R Core Team . (2018). R: A language and environment for statistical computing. R Foundation for Statistical Computing. https://www.R-project.org/

[hbm26342-bib-0080] Rittman, T. , Borchert, R. , Jones, S. , van Swieten, J. , Borroni, B. , Galimberti, D. , Masellis, M. , Tartaglia, M. C. , Graff, C. , Tagliavini, F. , Frisoni, G. B. , Laforce, R., Jr. , Finger, E. , Mendonça, A. , Sorbi, S. , Rohrer, J. D. , Rowe, J. B. , Afonso, S. , Almeida, M. R. , … Zulaica, M. (2019). Functional network resilience to pathology in presymptomatic genetic frontotemporal dementia. Neurobiology of Aging, 77, 169–177. 30831384 10.1016/j.neurobiolaging.2018.12.009 PMC6491498

[hbm26342-bib-0081] Roy, D. S. , Zhang, Y. , Halassa, M. M. , & Feng, G. (2022). Thalamic subnetworks as units of function. Nature Neuroscience, 25(2), 140–153. 35102334 10.1038/s41593-021-00996-1 PMC9400132

[hbm26342-bib-0082] Seeley, W. W. (2017). Mapping neurodegenerative disease onset and progression. Cold Spring Harbor Perspectives in Biology, 9(8), a023622. 28289062 10.1101/cshperspect.a023622 PMC5538416

[hbm26342-bib-0083] Seeley, W. W. , Crawford, R. K. , Zhou, J. , Miller, B. L. , & Greicius, M. D. (2009). Neurodegenerative diseases target large‐scale human brain networks. Neuron, 62(1), 42–52. 19376066 10.1016/j.neuron.2009.03.024 PMC2691647

[hbm26342-bib-0084] Sheline, Y. I. , & Raichle, M. E. (2013). Resting state functional connectivity in preclinical Alzheimer's disease. Biological Psychiatry, 74(5), 340–347. 23290495 10.1016/j.biopsych.2012.11.028 PMC3537262

[hbm26342-bib-0085] Simon, N. , Friedman, J. , Hastie, T. , & Tibshirani, R. (2011). Regularization paths for Cox's proportional hazards model via coordinate descent. Journal of Statistical Software, 39(5), 1–13. http://www.jstatsoft.org/v39/i05/ 10.18637/jss.v039.i05 PMC4824408 27065756

[hbm26342-bib-0086] Smith, S. M. , Beckmann, C. F. , Andersson, J. , Auerbach, E. J. , Bijsterbosch, J. , Douaud, G. , Duff, E. , Feinberg, D. A. , Griffanti, L. , Harms, M. P. , Kelly, M. , Laumann, T. , Miller, K. L. , Moeller, S. , Petersen, S. , Power, J. , Salimi‐Khorshidi, G. , Snyder, A. Z. , Vu, A. T. , … WU‐Minn HCP Consortium . (2013). Resting‐state fMRI in the human connectome project. NeuroImage, 80, 144–168. 23702415 10.1016/j.neuroimage.2013.05.039 PMC3720828

[hbm26342-bib-0087] Smith, S. M. , Vidaurre, D. , Beckmann, C. F. , Glasser, M. F. , Jenkinson, M. , Miller, K. L. , Nichols, T. E. , Robinson, E. C. , Salimi‐Khorshidi, G. , Woolrich, M. W. , Barch, D. M. , Uğurbil, K. , & van Essen, D. C. (2013). Functional connectomics from resting‐state fMRI. Trends in Cognitive Sciences, 17(12), 666–682. 24238796 10.1016/j.tics.2013.09.016 PMC4004765

[hbm26342-bib-0088] Smyser, C. D. , Inder, T. E. , Shimony, J. S. , Hill, J. E. , Degnan, A. J. , Snyder, A. Z. , & Neil, J. J. (2010). Longitudinal analysis of neural network development in preterm infants. Cerebral Cortex, 20(12), 2852–2862. 20237243 10.1093/cercor/bhq035 PMC2978240

[hbm26342-bib-0089] Spooner, A. , Chen, E. , Sowmya, A. , Sachdev, P. , Kochan, N. A. , Trollor, J. , & Brodaty, H. (2020). A comparison of machine learning methods for survival analysis of high‐dimensional clinical data for dementia prediction. Scientific Reports, 10(1), 20410. 33230128 10.1038/s41598-020-77220-w PMC7683682

[hbm26342-bib-0090] Steele, J. C. , Richardson, J. C. , & Olszewski, J. (1964). Progressive Supranuclear palsy: A heterogeneous degeneration involving the brain stem, basal ganglia and cerebellum with vertical gaze and pseudobulbar palsy, nuchal dystonia and dementia. Archives of Neurology, 10(4), 333. 14107684 10.1001/archneur.1964.00460160003001

[hbm26342-bib-0091] Stern, Y. , Arenaza‐Urquijo, E. M. , Bartrés‐Faz, D. , Belleville, S. , Cantilon, M. , Chetelat, G. , Ewers, M. , Franzmeier, N. , Kempermann, G. , Kremen, W. S. , Okonkwo, O. , Scarmeas, N. , Soldan, A. , Udeh‐Momoh, C. , Valenzuela, M. , & Vemuri, P. (2020). Whitepaper: Defining and investigating cognitive reserve, brain reserve, and brain maintenance. Alzheimer's Dementia, 16(9), 1305–1311. 10.1016/j.jalz.2018.07.219 PMC6417987 30222945

[hbm26342-bib-0092] Tingley, D. , Yamamoto, T. , Hirose, K. , Keele, L. , & Imai, K. (2014). Mediation: *R* package for causal mediation analysis. Journal of Statistical Software, 59(5), 1–38. http://www.jstatsoft.org/v59/i05/

[hbm26342-bib-0093] Tsvetanov, K. A. , Gazzina, S. , Jones, P. S. , Swieten, J. , Borroni, B. , Sanchez‐Valle, R. , Moreno, F. , Laforce, R Jr. , Graff, C. , Synofzik, M. , Galimberti, D. , Masellis, M. , Tartaglia, M. C. , Finger, E. , Vandenberghe, R. , de Mendonça, A. , Tagliavini, F. , Santana, I. , Ducharme, S. , … Genetic FTD Initiative, GENFI . (2021). Brain functional network integrity sustains cognitive function despite atrophy in presymptomatic genetic frontotemporal dementia. Alzheimer's Dementia, 17(3), 500–514. 10.1002/alz.12209 PMC7611220 33215845

[hbm26342-bib-0094] Tsvetanov, K. A. , Henson, R. N. A. , Tyler, L. K. , Davis, S. W. , Shafto, M. A. , Taylor, J. R. , Williams, N. , Cam‐CAN , & Rowe, J. B. (2015). The effect of ageing on f mri: Correction for the confounding effects of vascular reactivity evaluated by joint f mri and meg in 335 adults. Human Brain Mapping, 36(6), 2248–2269. 25727740 10.1002/hbm.22768 PMC4730557

[hbm26342-bib-0095] VandeVrede, L. , Ljubenkov, P. A. , Rojas, J. C. , Welch, A. E. , & Boxer, A. L. (2020). Four‐repeat Tauopathies: Current management and future treatments. Neurotherapeutics, 17(4), 1563–1581. 32676851 10.1007/s13311-020-00888-5 PMC7851277

[hbm26342-bib-0096] Voevodskaya, O. , Simmons, A. , Nordenskjöld, R. , Kullberg, J. , Ahlström, H. , Lind, L. , Wahlund, L‐O., Larsson, E‐M., & Westman, E; Alzheimer's Disease Neuroimaging Initiative (2014). The effects of intracranial volume adjustment approaches on multiple regional MRI volumes in healthy aging and Alzheimer's disease. Frontiers in Aging Neuroscience, 6, 264. 10.3389/fnagi.2014.00264/abstract PMC4188138 25339897

[hbm26342-bib-0097] Wear, H. J. , Wedderburn, C. J. , Mioshi, E. , Williams‐Gray, C. H. , Mason, S. L. , Barker, R. A. , & Hodges, J. R. (2008). The Cambridge Behavioural inventory revised. Dementia & Neuropsychologia, 2(2), 102–107. 29213551 10.1590/S1980-57642009DN20200005 PMC5619578

[hbm26342-bib-0098] Whiteside, D. J. , Jones, P. S. , Ghosh, B. C. P. , Coyle‐Gilchrist, I. , Gerhard, A. , Hu, M. T. , Klein, J. C. , Leigh, P. N. , Church, A. , Burn, D. J. , Morris, H. R. , Rowe, J. B. , & Rittman, T. (2021). Altered network stability in progressive supranuclear palsy. Neurobiology of Aging, 107, 109–117. 34419788 10.1016/j.neurobiolaging.2021.07.007 PMC8599965

[hbm26342-bib-0099] Whitwell, J. L. , Avula, R. , Master, A. , Vemuri, P. , Senjem, M. L. , Jones, D. T. , Jack, C. R., Jr. , & Josephs, K. A. (2011). Disrupted thalamocortical connectivity in PSP: A resting‐state fMRI, DTI, and VBM study. Parkinsonism & Related Disorders, 17(8), 599–605. 21665514 10.1016/j.parkreldis.2011.05.013 PMC3168952

[hbm26342-bib-0100] Whitwell, J. L. , Jack, C. R. , Boeve, B. F. , Parisi, J. E. , Ahlskog, J. E. , Drubach, D. A. , Senjem, M. L. , Knopman, D. S. , Petersen, R. C. , Dickson, D. W. , & Josephs, K. A. (2010). Imaging correlates of pathology in corticobasal syndrome. Neurology, 75(21), 1879–1887. 21098403 10.1212/WNL.0b013e3181feb2e8 PMC2995388

[hbm26342-bib-0101] Winawer, J. , Horiguchi, H. , Sayres, R. A. , Amano, K. , & Wandell, B. A. (2010). Mapping hV4 and ventral occipital cortex: The venous eclipse. Journal of Vision, 10(5), 1. 10.1167/10.5.1 PMC3033222 20616143

[hbm26342-bib-0102] Yeo, B. T. T. , Krienen, F. M. , Sepulcre, J. , Sabuncu, M. R. , Lashkari, D. , Hollinshead, M. , Roffman, J. L. , Smoller, J. W. , Zöllei, L. , Polimeni, J. R. , Fischl, B. , Liu, H. , & Buckner, R. L. (2011). The organization of the human cerebral cortex estimated by intrinsic functional connectivity. Journal of Neurophysiology, 106(3), 1125–1165. 21653723 10.1152/jn.00338.2011 PMC3174820

[hbm26342-bib-0103] Yu, M. , Linn, K. A. , Cook, P. A. , Phillips, M. L. , McInnis, M. , Fava, M. , Trivedi, M. H. , Weissman, M. M. , Shinohara, R. T. , & Sheline, Y. I. (2018). Statistical harmonization corrects site effects in functional connectivity measurements from multi‐site fMRI data. Human Brain Mapping, 39(11), 4213–4227. 29962049 10.1002/hbm.24241 PMC6179920

[hbm26342-bib-0104] Zhang, J. , Wang, J. , Xu, X. , You, Z. , Huang, Q. , Huang, Y. , Guo, Q. , Guan, Y. , Zhao, J. , Liu, J. , Xu, W. , Deng, Y. , Xie, F. , & Li, B. (2023). In vivo synaptic density loss correlates with impaired functional and related structural connectivity in Alzheimer's disease. Journal of Cerebral Blood Flow and Metabolism, 0271678X2311537. 10.1177/0271678X231153730 PMC10196742 36718002

[hbm26342-bib-0105] Zhou, J. , Gennatas, E. D. , Kramer, J. H. , Miller, B. L. , & Seeley, W. W. (2012). Predicting regional neurodegeneration from the healthy brain functional connectome. Neuron, 73(6), 1216–1227. 22445348 10.1016/j.neuron.2012.03.004 PMC3361461

