# Supplementary material for: Network connectivity and structural correlates of survival in progressive supranuclear palsy and corticobasal syndrome
Source: Hum Brain Mapp. 2023 Jun 3;44(11):4239–55. doi: 10.1002/hbm.26342 (PMC10318264; doi:10.1002/hbm.26342)
Supplement: Supplementary file 1 — Data S1: Supporting Information [file HBM-44-4239-s001.docx]

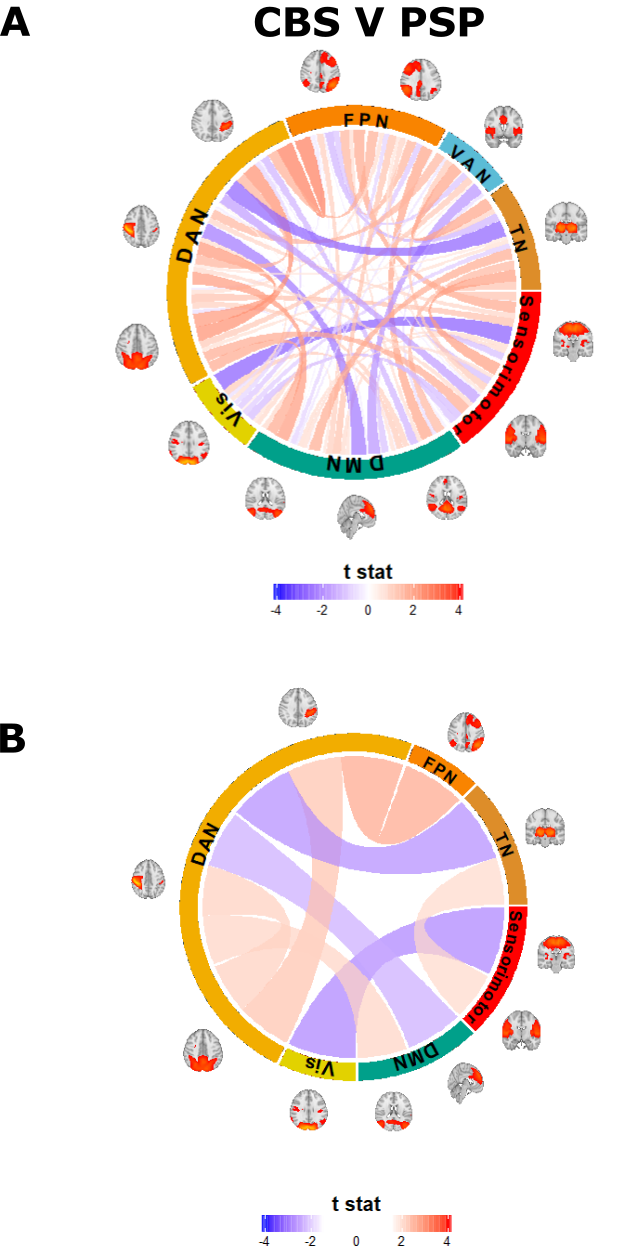


**Supplementary Figure 1:** Differences in between-network connectivity between PSP and CBS. Red links represent lower connectivity in CBS, and blue links lower connectivity in PSP. The bottom figure show only connections that show uncorrected significant differences (p < 0.05) between group differences after adjusting for age and sex.


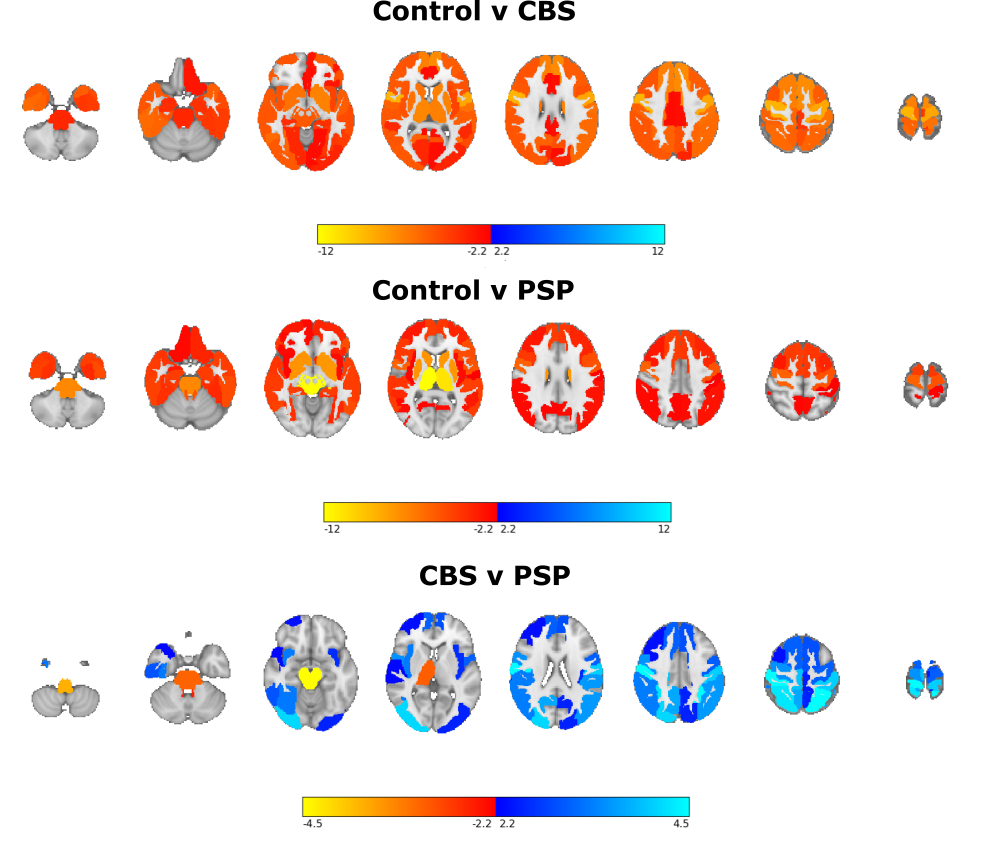


**Supplementary Figure 2:** Differences in cortical thickness and subcortical volume by group. T values are thresholded such that all parcels shown are significant after false discovery rate correction for multiple comparisons.

**
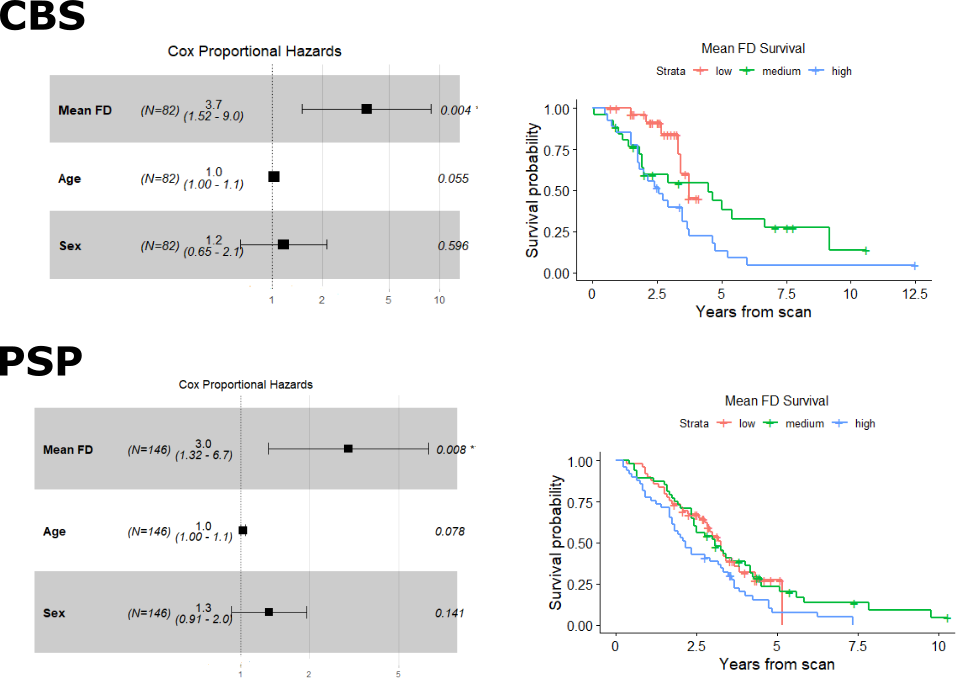
**

**Supplementary Figure 3:** Mean framewise displacement from resting state functional MRI is a significant predictor of survival in both PSP and CBS. Here all participants are considered prior to any exclusion for excess motion.


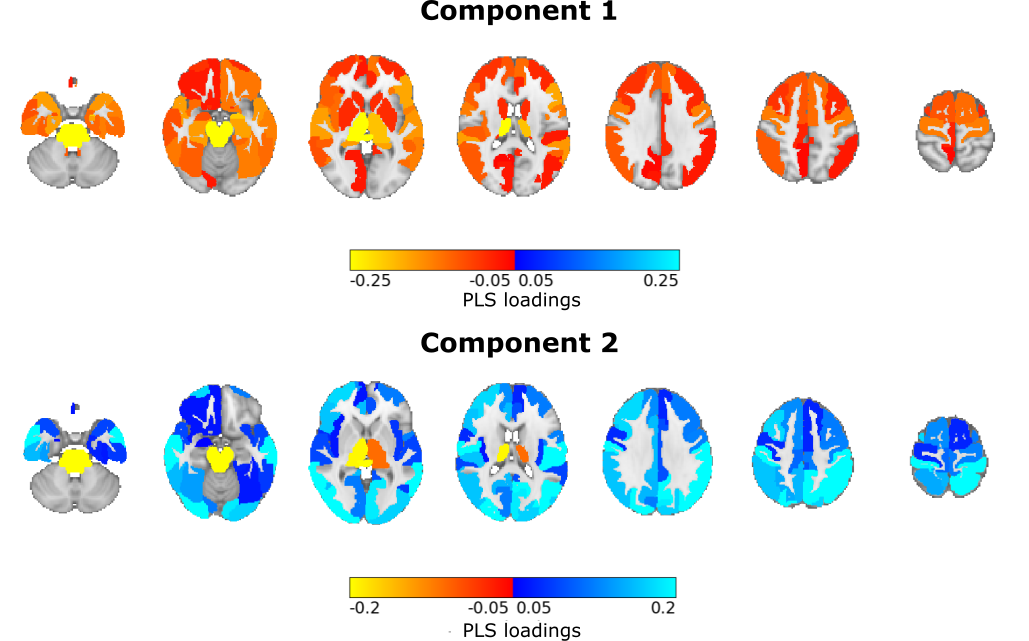


**Supplementary Figure 4:** Partial least squares regression weights for structural components predictive of survival.

**Supplementary table 1:** Motion parameters by group at baseline scan

|  | **Control** | **PSP** | **CBS** | **F** | **p** |
| --- | --- | --- | --- | --- | --- |
| **Mean framewise displacement** | 0.19 (0.12) | 0.18 (0.13) | 0.26 (0.22) | 7.5 | 0.0007 |
| **Median spike percentage** | 3.9 (2.6) | 3.5 (2.5) | 3.5 (3.5) | 0.5 | 0.59 |
| **Max spike percentage** | 14.2 (9.0) | 18.3 (11.2) | 13.8 (10.4) | 5.2 | 0.006 |
| **Max DVARS** | 7.8 (1.1) | 7.9 (1.1) | 7.6 (1.3) | 1.1 | 0.33 |
| **Mean DVARS** | 5.7 (0.5) | 5.6 (0.6) | 5.5 (0.8) | 2.1 | 0.13 |

**Supplementary table 2:**  Fixed effects for mixed linear models for different neuropsychological tests, with t and p-values for the years terms using Satterthwaite’s method

|  | **Intercept** | **years** | **t** | **P** |
| --- | --- | --- | --- | --- |
| **ACER** | 80.1 | -4.3 | -8.2 | 4x10^-12^ |
| **CBIR** | 42.2 | 8.0 | 5.1 | 5x10^-6^ |
| **PSPRS** | 32.6 | 6.8 | 10.6 | 2x10^-11^ |

**Supplementary Table 3: Stepwise regression for modelling progression in PSPRS**

| **Initial model/step** | **AIC** |
| --- | --- |
| Slope~ ICA1 + ICA2 +Baseline PSPRS + Total Grey Matter Volume + covariates | -11.9 |
| - ICA2 | -13.9 |
| -Total grey matter volume | -14 |
| Final Model | Slope – ICA1 + Baseline PSPRS + covariates |

**Supplementary Table 4: Stepwise regression for modelling progression in ACER**

| **Initial model/step** | **AIC** |
| --- | --- |
| Slope~ ICA1 + ICA2 +Baseline ACER + Total Grey Matter Volume + covariates | -46.1 |
| - ICA1 | -47.6 |
| Final Model | Slope – ICA2 + Baseline ACER + covariates |

**Supplementary Table 5: Stepwise regression for modelling progression in CBIR**

| **Initial model/step** | **AIC** |
| --- | --- |
| Slope~ ICA1 + ICA2 +Baseline CBIR + Total Grey Matter Volume + covariates | 8.1 |
| - CBIR | 6.2 |
| - Total Gray Matter Volume | 4.5 |
| Final Model | Slope –ICA1 + ICA2 + Baseline ACER + covariates |

**Supplementary table 6:** PLSR coefficients for a one component PLSR Cox model

| **Predictor** | **PLSR standardised coefficients** | **P value** |
| --- | --- | --- |
| PSPRS | 0.20 | 3.1 x 10^-8^ |
| CBIR | 0.10 | 5.1 x 10^-4^ |
| ACER | -0.056 | 0.058 |
| Age | 0.067 | 0.013 |

**Supplementary table 7:** PLSR standardised coefficients for a one component model in participants with PSP using all predictors.

| **Predictor** | **PLSR weight** |
| --- | --- |
| ***Positive weights*** |  |
| PSPRS | 0.32 |
| ***Negative weights*** |  |
| Pons | -0.26 |
| Midbrain | -0.25 |
| Left superior temporal gyrus | -0.21 |
| Left thalamus | -0.19 |
| Right thalamus | -0.18 |
| Right superior temporal gyrus | -0.17 |
| Left amygdala | -0.16 |
| Right frontoparietal-thalamic connectivity | -0.15 |

**Supplementary table 8:** PLSR standardised coefficients for a one component model in participants with CBS using all predictors.

| **Predictor** | **PLSR weight** |
| --- | --- |
| ***Positive weights*** |  |
| Visual-posterior default model connectivity | 0.20 |
| PSPRS | 0.19 |
| ***Negative weights*** |  |
| Right thalamus | -0.24 |
| Midbrain | -0.20 |
| Pons | -0.20 |
| Right hippocampus | -0.16 |
| Left amygdala | -0.16 |
| Left hippocampus | -0.16 |
| Right precentral gyrus | -0.15 |
| Right inferior temporal gyrus | -0.15 |
| Dorsal attention-visual connectivity | -0.15 |
